# New Methods for the Synthesis of Spirocyclic Cephalosporin Analogues

**DOI:** 10.3390/molecules26196035

**Published:** 2021-10-05

**Authors:** Alan X. Zhao, Louise E. Horsfall, Alison N. Hulme

**Affiliations:** 1 EaStChem School of Chemistry, The University of Edinburgh, Joseph Black Building, David Brewster Road, Edinburgh EH9 3FJ, UK; s1117565@sms.ed.ac.uk; 2 Institute of Quantitative Biology, Biochemistry, and Biotechnology, School of Biological Science, The University of Edinburgh, Roger Land Building, Alexander Crum Brown Road, Edinburgh EH9 3FF, UK; louise.horsfall@ed.ac.uk

**Keywords:** spiro-cyclisation, spiro-cephalosporin, Michael-type reaction

## Abstract

Spiro compounds provide attractive targets in drug discovery due to their inherent three-dimensional structures, which enhance protein interactions, aid solubility and facilitate molecular modelling. However, synthetic methodology for the spiro-functionalisation of important classes of penicillin and cephalosporin β-lactam antibiotics is comparatively limited. We report a novel method for the generation of spiro-cephalosporin compounds through a Michael-type addition to the dihydrothiazine ring. Coupling of a range of catechols is achieved under mildly basic conditions (K_2_CO_3_, DMF), giving the stereoselective formation of spiro-cephalosporins (d.r. 14:1 to 8:1) in moderate to good yields (28−65%).

## 1. Introduction

Spiro compounds (or spirocycles) are rigid scaffolds that consist of at least two rings fused through a single atom, known as the spiro atom. The structure of spirocycles was first discovered by Von Baeyer in the late 1890s [1]. Spiro compounds are an important class of molecule, and spirocycles are found in a number of different natural products with pronounced biological activities. Griseofulvin, for example, is a spirocyclic natural product generated by the fungus *Penicillium griseofulvum* that has been used clinically to treat dermatophytosis (ringworm) [2]. 

In recent years, spirocycles have emerged as attractive synthetic targets in drug discovery. Growing efforts have been made to employ spirocyclic scaffolds as core or periphery structures in drug synthesis, and many show promising biological activities as cholesterol absorption inhibitors [3,4,5], anti-Parkinsonian agents [6,7,8], anti-Alzheimer’s agents [9,10,11], antiviral agents [12,13,14,15], antibacterial agents [16,17,18], β-lactamase inhibitors [19] and anti-cancer agents [20,21,22,23]. The inherent three-dimensional nature of spiro compounds provides better protein interactions as compared to their planar (hetero)aromatic counterparts [24]. Moreover, spirocyclic scaffolds have relatively rigid structures and possess a limited number of well-defined conformations, which is an advantage when using in silico docking to optimise interactions between drugs and protein targets computationally [25]. The inherent sp^3^ nature of spirocycles, means they are also generally more water soluble than the corresponding (hetero)aromatic systems [26], and the introduction of a spirocycle can be used to modulate the water solubility of compounds of interest [24,27]. 

Nevertheless, spirocycles are considered a synthetic challenge, mainly due to the difficulty in synthesising quaternary carbon centres, as well as controlling the stereochemistry [28,29]. Multiple synthetic strategies have been employed to synthesise spirocycles including alkylation methods, cycloaddition approaches, rearrangement approaches, ring closure methods and radical cyclisation approaches [28,29,30,31].

**Figure 1:**
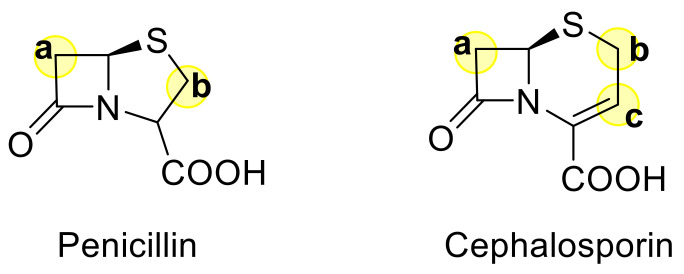
Modification sites for attaching spirocyclic moieties.

Among the many possible spirocyclic structures, spirocyclic β-lactams have drawn increasing attention, not only because they exhibit desirable antibacterial properties [32,33,34], but also because they show other interesting activities as enzyme inhibitors [3,19,35,36] and antivirals [37]. Multiple reaction sites can be utilised to modify the penicillin- or cephalosporin-based β-lactam antibiotics with spirocyclic moieties without altering their β-lactam core structures. Figure 1 shows that the spirocycles can be either fused directly onto the β-lactam ring (site **a**) or attached to the thiazolidine or dihydrothiazine ring (sites **b** and **c**). 

A number of different methods to achieve spiro-modification at site **a** have been reported including: (i) 1,3-dipolar cycloaddition approaches [38,39]; (ii) a phosphane-catalysed [3+2] cycloaddition approach [40]; and (iii) a rhodium-catalysed cyclopropanation approach [19]. However, methodologies for spiro-modifications at site **b** and **c** are rather limited, and mostly rely on 1,3-dipolar cycloadditon reactions [41]. In this work, we report a novel synthetic strategy to synthesise spirocyclic cephalosporin analogues at site **c** through a Michael-type reaction. 

## 2. Results and Discussion

**Scheme 1:**
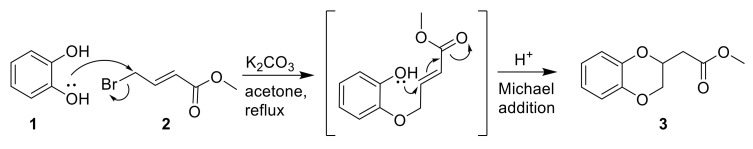
Generation of benzodioxane **3** via a Michael addition reaction.

To achieve spiro-modification at site **c** of cephalosporin, catechols were used. Catechols are commonly found in natural products, insecticides and pharmaceuticals. They have well-documented antioxidant properties, acting as structural units in many bronchodilator, adrenergic, antioxidant, anti-Parkinsonian and anti-hypertensive drugs [42]. Catechols are important building blocks in organic synthesis and are frequently used as nucleophiles in substitution reactions due to the nucleophilicity of the catechol monoanion; catechols have also been used as nucleophilic catalysts for peptide bond formation [43]. Furthermore, catechols can undergo Michael addition to α,β-unsaturated carbonyl compounds to generate six-membered heterocyclic compounds under mild basic conditions. Cabbidu et al. reported a method to synthesise benzodioxane **3** by reacting pyrocatechol **1** with methyl 4-bromocrotonate **2** in the presence of K_2_CO_3_ (Scheme 1) [44]. The reaction is thought to proceed via nucleophilic substitution of the bromine, followed by Michael addition to yield the desired product **3**. 

Inspired by the work by Cabbidu et. al, we decided to take a similar approach, utilising the α,β-unsaturated carbonyl moiety within the cephalosporin structure to react with catechols for the generation of spiro-cephalosporin compounds through Michael addition reactions.

### 2.1. Reaction Scope for Generating Spiro-Cephalosporins

**Scheme 2:**
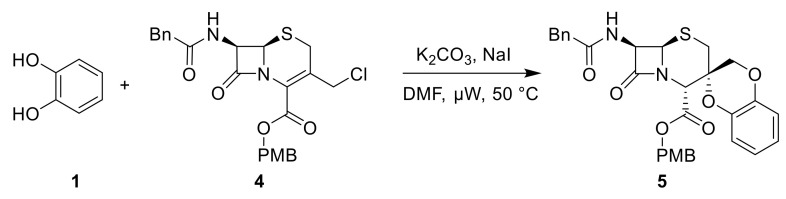
Generation of benzodioxane **3** via Michael addition reaction.

The spiro-cyclisation reaction was first conducted by reacting commercially available cephalosporin derivative **4** with pyrocatechol **1** in the presence of K_2_CO_3_ [45], under microwave irradiation at 50 °C to accelerate the reaction, as shown in Scheme 2.

**Figure 2:**
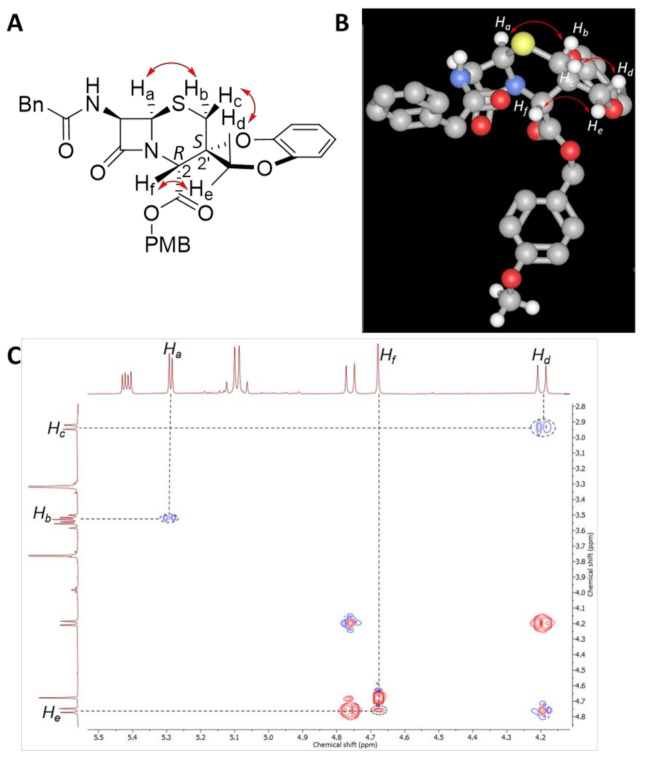
Structure of spiro-cephalosporin **5** in (**A**) 2-D and (**B**) 3-D; (**C**) the NOESY spectrum.

Spiro-cephalosporin **5** was isolated from the reaction mixture by column chromatography as a single diastereomer in moderate yield (40%). Stereochemical assignment was achieved using the NOESY spectrum of **5** (Figure 2), which shows the interaction of the ring junction proton *H_a_* with methylene proton *H_b_*, and the second methylene proton *H_c_* with the axial proton *H_d_* on the benzodioxane ring. An nOe interaction is also observed between the equatorial proton *H_e_* on the benzodioxane ring and *H_f_*, indicating these two protons are on the same face. Therefore, the C-2′ and C-2 stereocentres in product **5** are determined as *S* and *R* configurations, as shown in Figure 2A (2D view) and B (3D view). 

To expand further the scope of the spiro-cyclisation reaction, a number of different catechols were screened under the same reaction conditions (Table 1). 

**Figure 3:**
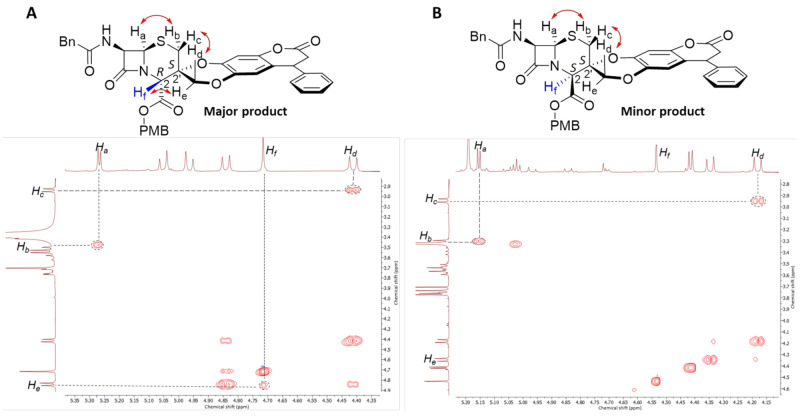
NOSEY spectra of (**A**) the major diastereomer **15** and (**B**) the minor diastereomer.

The spiro-cyclisation reactions with dihydroxy coumarins (Table 1, entries 1–3) and flavonoids (Table 1, entries 4–6) were explored. Reactions were monitored by HPLC, and it was found that diastereomers were formed in the reactions of coumarins **7** and **8**, and flavonoid **9** with diastereomeric ratios of 14:1, 9:1, and 12:1, respectively. For entry 3, the two diastereomers were successfully isolated and the characteristic NMR peaks were assigned to allow NOESY analysis to identify their stereochemistry. Figure 3A shows the NOESY spectra for the major diastereomer **15**, in which nOe interactions are observed between *H_a_* and *H_b_*, *H_c_* and *H_d_*, and *H_e_* and *H_f_*. Therefore, the configurations of the newly generated chiral centres at the C-2′ and C-2 positions were, again, determined to be *S* and *R,* respectively. In contrast, no interaction is observed between *H_e_* and *H_f_* for the minor diastereomer (Figure 3B), suggesting these two protons are not on the same face and, thus the minor diastereomer has a (2′*S*,2*S*) configuration. For the other reactions, only the major diastereomer was isolated by column chromatography in each case and the stereochemistry was found to be the same as for the major diastereomer **15**, i.e., a (2′*S*,2*R*) configuration.

The reaction with flavonoid **10** which contains three adjacent hydroxyl groups was also studied (Table 1, entry 5). The reaction was found to be regioselective, as spiro-product **17** was the predominant product (see Section 2.3). Again, the stereochemistry at the C-2′ and C-2 positions of **17** was determined to be *S* and *R* configuration by NOESY NMR.

We also investigated the regioselectivity of spiro-cycloaddition when a flavonoid contains multiple hydroxyl groups. Quercetin **11** was reacted with cephalosporin **4** in the presence of K_2_CO_3_ (Table 1, entry 6). However, the reaction was unsuccessful, and no spiro-product **18** was isolated from the reaction mixture. From the LC-MS analysis, a mass of 753 Da was detected, which corresponds to the mass of an alkylated product. The most acidic phenol in quercetin is known to be the 7-OH on the flavonol core, thus the monoanion is most likely to form here. Reaction with cephalosporin **4** (see Section 2.3) gives an adduct which cannot proceed to spirocyclisation, resulting in the formation of an acyclic product. However, this putative alkylation product could not be isolated by column chromatography on silica gel, suggesting facile decomposition under mild acidic conditions. 

Finally, we tested the reaction with the polyphenol, ellagic acid **12** (Table 1, entry 7). The reaction proceeded successfully; two diastereomers were observed in the reaction mixture (HPLC ratio of 8:1). However, the yield of the reaction was poor (28%), suggesting that the steric bulk provided by the polycyclic core of ellagic acid disfavours formation of spiro-product **19**.

### 2.2. Functionalisation of Spiro-Cephalosporins

To provide functionally active spiro-cephalosporin compounds, the *p*-methoxybenzyl (PMB) protecting group was removed in the presence of trifluoroacetic acid (TFA) and anisole. The crude products were purified by column chromatography to give the carboxylic acids as single diastereomers in good yields (63−97%), as shown in Table 2. 

### 2.3. Proposed Mechanism

**Figure 4:**
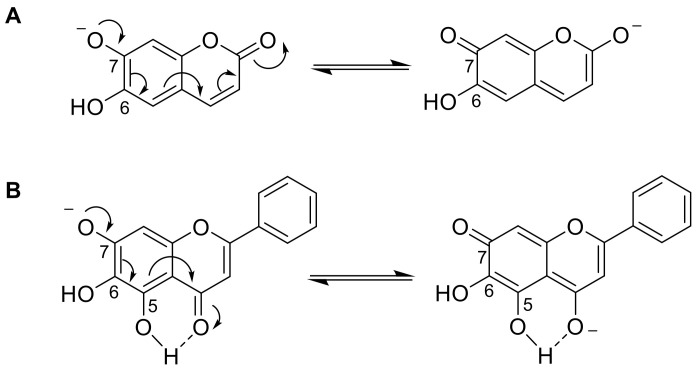
Resonance structures of (**A**) coumarin **6** and (**B**) flavonoid **10** under mild basic conditions.

For the 6,7-dihydroxycoumarins (**6**–**8**), the 7-OH (p*K_a_* = 7.5) is reported to be much more acidic than the 6-OH (p*K_a_* = 9.0) [46]. This difference in acidity can be explained by resonance stabilisation as shown in Figure 4A. Similarly, for flavonoids (**9** and **10**), the 7-OH (p*K_a_* = 7.7) is more acidic than the 6-OH (p*K_a_* = 9.0) due to resonance stabilisation [47], and, hence, can be deprotonated more easily under basic conditions (Figure 4B). In addition, for 5,6,7-trihydroxyflavonoid **10**, the 5-OH is found to be least acidic (p*K_a_* = 11.4) due to the formation of an intramolecular hydrogen bond with the adjacent carbonyl group (Figure 4B) [47,48]. Thus spiro-cyclisation can be initiated by the 7-OH and is completed by the adjacent 6-OH to give the observed regioselectivity in compound **17** shown in Table 1, entry 5. In contrast, the two hydroxyl groups in pyrocatechol **1** have a much higher p*K_a_* value (p*K_a_* = 9.3)[49] than the 7-OH in the dihydroxy coumarins and flavonoids, which cannot be easily deprotonated under mild basic conditions, resulting in a lower reaction yield (40%).

**Figure 5:**
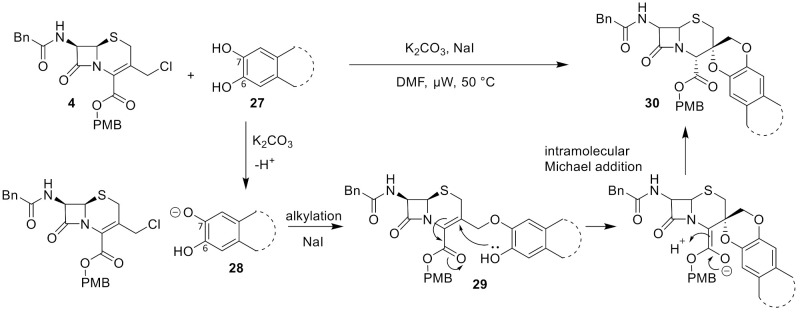
Proposed spiro-cyclisation mechanism for the formation of spiro-cephalosporin **30**.

A mechanism for the generation of the spiro-cephalosporin product **30** is therefore proposed as shown in Figure 5. Under mildly basic conditions, the more acidic 7-OH in catechol **27** is readily deprotonated to form the reactive phenolate ion **28**. The phenolate ion **28** is then alkylated by cephalosporin **4** in the presence of NaI to form intermediate **29**. The experimental p*Ka* of the phenol in mono-alkylated catechols is consistently reported to be several log units higher [50,51] than the most acidic phenol in their non-alkylated precursors, hence a second phenolate alkylation reaction is not favoured under these conditions. Rather, phenol **29** undergoes an intramolecular Michael addition to form the spiro-cephalosporin **30**, with initial attack on the convex face of the cephalosporin core, followed by protonation on the face opposite the newly-introduced, bulky catechol unit to set the observed (2′*S*,2*R*) stereochemistry.

## 3. Materials and Methods

### 3.1. General Information

All reagents were obtained from commercial suppliers and were used without further purification. Microwave reactions were performed using Biotage Initiator+. Flash chromatography was carried out using Merck Kieselgel 60 (Merck 9385) under positive pressure. ^1^H and ^13^C NMR spectra were obtained on a Bruker AVA 500 instrument, using TMS as a reference and residual solvent as an internal standard. The data are presented as follows: chemical shift (in ppm on the δ scale relative to δTMS = 0), integration, multiplicity, coupling constant and interpretation. ^1^H and ^13^C spectra for all new compounds are presented in the SI file and raw data in the accompanying data deposit. Electrospray ionisation (ESI) mass spectra were obtained on a Bruker microTOF II instrument. Analytical Reverse Phase HPLC (Analytical RP-HPLC) was conducted on a Waters^®^ 600 (100 µL) system using a 717plus autosampler and 996 PDA detector (190 to 800 nm) equipped with a Phenomenex^®^ SphereClone ODS(2), 5 µm, 100 × 4.6 mm column for HPLC (method 1) or with a Phenomenex Luna C18(2), 5 µm, 250 × 4.6 mm column for HPLC (method 2). A binary solvent system was used A = water (0.1% TFA), B = MeCN (0.1% TFA) at a flow rate of 1.00 mL∙min^−1^; and the column was maintained at 30 ± 1 °C. The HPLC (method 1) was a linear gradient from 0 min (95A:5B) to 10 min (5A:95B), isocratic from 10 min to 12 min (5A:95B), before recovery of the initial conditions over 3 min and equilibration over 5 min, giving a total run time of 20 min. The HPLC (method 2) was a linear gradient from 0 min (95A:5B) to 30 min (5A:95B), isocratic from 30 min to 35 min (5A:95B), before recovery of the initial conditions over 5 min and equilibration over 10 min, giving a total run time of 50 min.

### 3.2. Typical Procedure for the Spiro-Cyclisation Reaction

Pyrocatechol **1** (30.0 mg, 0.27 mmol), Cepham ester **4** (0.13 g, 0.27 mmol), NaI (4.08 mg, 0.03 mmol) and K_2_CO_3_ (0.11 g, 0.82 mmol) were dissolved in DMF (3 mL). The reaction mixture was stirred at 50 °C under microwave irradiation for 50 min. The reaction mixture was concentrated in vacuo then diluted with EtOAc (20 mL). The solution was washed with water (20 mL), Na_2_SO_3_ (20 mL, 5% aq) and brine (20 mL, sat aq), then dried (MgSO_4_) and concentrated in vacuo. The crude product was purified with column chromatography (hexane:EtOAc, 3:1) to give the desired product **5** [45].

### 3.3. Characterisation of Spiro-Cephalosporin Products 

(4-Methoxyphenyl)methyl (2′*S*,2*R*,6*R*,7*R*)-8-oxo-7-(2-phenylacetamido)-5-thia-1-azaspiro[1′,4′-benzodioxine-3,2′-bicyclo[4.2.0]octane]-2-carboxylate (**5**): 61.2 mg (40%); R_t_ (method 1) = 14.52 min; R_f_ (hexane:EtOAc, 3:1) = 0.16; mp 157 – 160 °C; IR 2920 (NH), 1770 (C=O), 1734 (C=O), 1683 (C=O); ^1^H NMR δ (500 MHz, DMSO-*d_6_*) 9.21 (1H, d, *J* = 8.5 Hz, NH), 7.34 – 7.22 (7H, m, Ar*H*), 6.97 – 6.83 (5H, m, Ar*H*), 6.64 (1H, dd, *J* = 7.9, 1.7 Hz, Ar*H*), 5.42 (1H, dd, *J* = 8.5, 4.3 Hz, NHC*H*), 5.29 (1H, dd, *J* = 4.3 Hz, SC*H*N), 5.11 (1H, d, *J* = 11.8 Hz, OC*H_A_*H_B_Ar), 5.08 (1H, d, *J* = 11.8 Hz, OCH_A_*H_B_*Ar), 4.76 (1H, d, *J* = 12.1 Hz, ArO*H_C_*H_D_), 4.68 (1H, s, NC*H*COO), 4.20 (1H, d, *J* = 12.1 Hz, ArOH_C_*H_D_*), 3.76 (3H, s, C*H_3_*), 3.57 (1H, d, *J* = 14.1 Hz, COC*H_E_*H_F_), 3.53 (1H, d, *J* = 13.7 Hz, SC*H_G_*H_H_), 3.52 (1H, d, *J* = 14.1 Hz, COCH_E_*H_F_*), 2.93 (1H, d, *J* = 13.7 Hz, SCH_G_*H_H_*). ^13^C NMR δ (126 MHz, DMSO-*d*_6_) 171.04 (C), 166.98 (C), 165.57 (C), 159.87 (C), 142.13 (C), 141.36 (C), 136.13 (C), 130.96 (2 × CH), 129.46 (2 × CH), 128.73 (2 × CH), 127.32 (C), 127.00 (CH), 122.77 (CH), 122.39 (CH), 117.84 (CH), 117.64 (CH), 114.23 (2 × CH), 70.81 (C), 67.26 (CH_2_), 65.86 (CH_2_), 59.44 (CH), 55.62 (CH_3_), 55.58 (CH), 52.76 (CH), 42.28 (CH_2_), 26.91 (CH_2_); *m/z* (ESI+, MeCN) 583 ([M+Na]^+^, 5%), 578 ([M+NH_4_]^+^, 100), 561 ([M+H]^+^, 30); HRMS (ESI+, MeCN) [M+H]^+^ found 561.1687, C_30_H_29_N_2_O_7_S requires 561.1690. 

(4-Methoxyphenyl)methyl (2′*S*,2*R*,6*R*,7*R*)-7′,8-dioxo-7-(2-phenylacetamido)-3′,7′-dihydro-5-thia-1-azaspiro[1′,4′-dioxino-3,2′-bicyclo[4.2.0]octane]-2-carboxylate (**13**): 210 mg (59%); R_t_ (method 1) = 12.39 min; R_f_ (EtOAc:DCM, 1:5) = 0.34; mp 100-103 °C; IR 3315 (NH), 1726 (C=O), 1681 (C=O), 1512 (C=C); ^1^H NMR δ (601 MHz, DMSO-*d_6_*) 9.21 (1H, d, *J* = 8.5 Hz, N*H*), 7.79 (1H, d, *J* = 9.5 Hz, COC*H*=CH), 7.32 – 7.22 (7H, m, Ar*H*), 7.08 (1H, s, Ar*H*), 6.96 (1H, s, Ar*H*), 6.86 (2H, d, *J* = 8.7 Hz, Ar*H*), 6.36 (1H, d, *J* = 9.5 Hz, COCH=C*H*), 5.42 (1H, dd, *J* = 8.5, 4.2 Hz, NHC*H*), 5.30 (1H, d, *J* = 4.2 Hz, SC*H*N), 5.13 (1H, d, *J* = 11.7 Hz, OC*H_A_*H_B_Ar), 5.03 (1H, d, *J* = 11.7 Hz, OCH_A_*H_B_*Ar), 4.88 (1H, d, *J* = 12.3 Hz, ArOC*H_C_*H_D_), 4.67 (1H, s, NC*H*COO), 4.32 (1H, d, *J* = 12.2 Hz, ArOCH_C_*H_D_*), 3.73 (3H, s, C*H_3_*), 3.56 (1H, d, *J* = 14.1 Hz, COC*H_E_*H_F_), 3.52 (1H, d, *J* = 13.8 Hz, SC*H_G_*H_H_), 3.51 (1H, d, *J* = 14.1 Hz, COCH_E_*H_F_*), 2.99 (1H, d, *J* = 13.8 Hz, SCH_G_*H_H_*); ^13^C NMR δ (151 MHz, DMSO-*d_6_*) 171.05 (C), 166.84 (C), 165.61 (C), 160.51 (C), 159.82 (C), 149.27 (C), 145.33 (C), 144.12 (CH), 138.13 (C), 136.11 (C), 131.05 (2 × CH), 129.47 (2 × CH), 128.74 (2 × CH), 127.10 (CH), 127.02 (C), 115.89 (CH), 114.50 (CH), 114.21 (2 × CH), 113.96 (C), 105.07 (CH), 70.90 (C), 67.38 (CH_2_), 66.34 (CH_2_), 59.53 (CH), 55.66 (CH_3_), 55.56 (CH), 52.60 (CH), 42.31 (CH_2_), 26.94 (CH_2_); *m/z* (ESI+, MeCN) 629 ([M+H]^+^, 100%); HRMS (ESI+, MeCN) [M+H]^+^ found 629.1597, C_33_H_29_N_2_O_9_S requires 629.1588.

(4-Methoxyphenyl)methyl (2′*S*,2*R*,6*R*,7*R*)-7′,8-dioxo-7-(2-phenylacetamido)-3′,7′,8′,9′,10′,11′-hexahydro-1′,4′,6′-trioxa-5-thia-1-azaspiro[bicyclo[4.2.0]octane-3,2′-tetraphene]-2-carboxylate (**14**): 420 mg (62%); R_t_ (method 1) =13.63 min; R_f_ (DCM:EtOAc, 8:1) = 0.25; mp 120 – 123 °C; IR 2934 (NH), 1626 (C=O), 1701 (C=O), 1578 (C=O), 1506 (C=C); ^1^H NMR δ (500 MHz, DMSO-*d_6_*) 9.21 (1H, d, *J* = 8.5 Hz, N*H*), 7.34 – 7.22 (5H, m, Ar*H*), 7.16 (2H, d, *J* = 8.7 Hz, Ar*H*), 7.07 (1H, s, C_5′_*H*), 6.88 (1H, s, C_12′_*H*), 6.81 (2H, d, *J* = 8.7 Hz, Ar*H*), 5.44 (1H, dd, *J* = 8.5, 4.2 Hz, NHC*H*), 5.32 (1H, d, *J* = 4.2 Hz, SC*H*N), 5.19 (1H, d, *J* = 12.1 Hz, OC*H_A_*H_B_Ar), 5.04 (1H, d, *J* = 12.1 Hz, OCH_A_*H_B_*Ar), 4.86 (1H, d, *J* = 12.2 Hz, ArOC*H_C_*H_D_), 4.74 (1H, s, NC*H*CO), 4.32 (1H, d, *J* = 12.2 Hz, ArOCH_C_*H_D_*), 3.71 (3H, s, C*H*_3_), 3.57 (1H, d, *J* = 14.1 Hz, COC*H_E_*H_F_), 3.56 (1H, d, *J* = 13.8 Hz, SC*H_G_*H_H_), 3.52 (1H, d, *J* = 14.1 Hz, COCH_E_*H_F_*), 3.01 (1H, d, *J* = 13.8 Hz, SCH_G_*H_H_*), 2.60 – 2.54 (1H, m, C_11′_*H*), 2.42 – 2.37 (3H, m, C_8′_*H*_2_, C_11′_*H*), 1.72 – 1.71 (4H, m, C_9′_*H*_2_C_10′_*H*_2_); ^13^C NMR δ (126 MHz, DMSO-*d_6_*) 171.05 (C), 166.97 (C), 165.64 (C), 161.11 (C), 159.60 (C), 147.31 (C), 147.01 (C), 144.07 (C), 138.14 (C), 136.11 (C), 130.17 (2 × CH), 129.47 (2 × CH), 128.74 (2 × CH), 127.27 (C), 127.02 (CH), 121.32 (C), 115.15 (C), 113.94 (2 × CH), 111.58 (CH), 104.83 (CH), 70.98 (C), 67.12 (CH_2_), 66.32 (CH_2_), 59.50 (CH), 55.73 (CH), 55.46 (CH_3_), 52.66 (CH), 42.32 (CH_2_), 26.96 (CH_2_), 25.13 (CH_2_), 24.11 (CH_2_), 21.57 (CH_2_), 21.26 (CH_2_); *m/z* (ESI+, MeCN) 705 ([M+Na]^+^, 63%), 683 ([M+H]^+^, 94); HRMS (ESI+, MeCN) [M+H]^+^ found 683.2087, C_37_H_35_N_2_O_9_S requires 683.2058. Minor diastereomer: R_t_ (method 1) =13.34 min; ^1^H NMR δ (500 MHz, DMSO-*d_6_*) diagnostic peaks: 9.12 (1H, d, *J* = 8.2 Hz, N*H*), 5.41 (1H, dd, *J* = 8.2, 4.3 Hz, NHC*H*), 5.19 (1H, d, *J* = 4.3 Hz, SC*H*N), 4.63 (1H, s, NC*H*CO), 3.77 (3H, s, C*H_3_*).

(4-Methoxyphenyl)methyl (2′*S*,2*R*,6*R*,7*R*)-7′,8-dioxo-9′-phenyl-7-(2-phenylacetamido)-3′,7′-dihydro-5-thia-1-azaspiro[1′,4′-dioxino-3,2’-bicyclo[4.2.0]octane]-2-carboxylate (**15**): 290 mg (54%); R_t_ (method 1) = 15.47 min; R_f_ (DCM:EtOAc, 6:1) = 0.51; mp 165 – 168 °C; IR 3120 (NH), 1762 (C=O), 1701 (C=O), 1514 (C=C); ^1^H NMR δ (500 MHz, DMSO-*d_6_*) 9.20 (1H, d, *J* = 8.5 Hz, N*H*), 7.60 – 7.58 (3H, m, Ar*H*), 7.46 – 7.44 (2H, m, Ar*H*), 7.33 – 7.24 (6H, m, Ar*H*), 7.07 (2H, d, *J* = 8.6 Hz, Ar*H*), 6.75 (1H, s, Ar*H*), 6.67 (2H, *J* = 8.6 Hz, Ar*H*), 6.32 (1H, s, =C*H*), 5.40 (1H, dd, *J* = 8.5, 4.2 Hz, NHC*H*), 5.27 (1H, d, *J* = 4.2 Hz, SC*H*N), 5.05 (1H, d, *J* = 12.1 Hz, OC*H_A_*H_B_Ar), 4.97 (1H, d, *J* = 12.1 Hz, OCH_A_*H_B_*Ar), 4.84 (1H, d, *J* = 12.2 Hz, ArOC*H_C_*H_D_), 4.72 (1H, s, NC*H*COO), 4.41 (1H, d, *J* = 12.2 Hz, ArOCH_C_*H_D_*), 3.70 (3H, s, C*H_3_*), 3.57 (1H, d, *J* = 14.1 Hz, COC*H_E_*H_F_Ar), 3.51 (1H, d, *J* = 14.1 Hz, COCH_E_*H_F_*Ar), 3.41 (1H, d, *J* = 13.9 Hz, SC*H_G_*H_H_), 2.94 (1H, d, *J* = 13.9 Hz, SCH_G_*H_H_*); ^13^C NMR δ (126 MHz, DMSO-*d_6_*) 171.07 (C), 166.63 (C), 165.58 (C), 160.24 (C), 159.64 (C), 154.89 (C), 149.44 (C), 145.74 (C), 138.26 (C), 136.08 (C), 135.19 (C), 130.32 (CH), 130.19 (2 × CH), 129.46 (4 × CH), 128.73 (2 × CH), 128.70 (2 × CH), 127.16 (C), 127.01 (CH), 114.04 (3 × CH), 113.82 (C), 113.45 (CH), 105.87 (CH), 71.26 (C), 67.28 (CH_2_), 66.41 (CH_2_), 59.53 (CH), 55.63 (CH), 55.58 (CH_3_), 52.84 (CH), 42.30 (CH_2_), 26.78 (CH_2_); *m/z* (ESI+, MeCN) 727 ([M+Na]^+^, 68%), 722 ([M+NH_4_]^+^, 14), 705 ([M+H]^+^, 100); HRMS (ESI+, MeCN) [M+H]^+^ found 705.1896, C_39_H_33_N_2_O_9_S requires 705.1901. Minor diastereomer: R_t_ (method 1) = 15.24 min; ^1^H NMR δ (500 MHz, DMSO-*d_6_*) diagnostic peaks: 9.10 (1H, d, *J* = 8.1 Hz, N*H*), 5.41 (1H, dd, *J* = 8.1, 4.3 Hz, NHC*H*), 5.19 (2H, s, OC*H_2_*Ar), 5.15 (1H, d, *J* = 4.3 Hz, SC*H*N), 4.54 (1H, s, NC*H*CO), 4.35 (1H, d, *J* = 12.2 Hz, ArOC*H_A_*H_B_), 4.18 (1H, d, *J* = 12.2 Hz, ArOCH_A_*H_B_*), 3.77 (3H, s, C*H_3_*), 3.58 (1H, *J* = 14.0 Hz, COC*H_C_*H_D_Ar), 3.52 (1H, *J* = 14.0 Hz, COCH_C_*H_D_*Ar), 3.29 (1H, d, *J* = 13.4 Hz, SC*H_E_*H_F_), 2.94 (1H, d, *J* = 13.4 Hz, SCH_E_*H_F_*).

(4-Methoxyphenyl)methyl (2′*S*,2*R,*6*R*,7*R*)-8,9′-dioxo-7′-phenyl-7-(2-phenylacetamido)-3′,9′-dihydro-5-thia-1-azaspiro[1′,4′-dioxino-3,2’-bicyclo[4.2.0]octane]-2-carboxylate (**16**): 67.3 mg (65%); R_t_ (method 1) = 15.52 min; R_f_ (DCM:EtOAc, 5:1) = 0.42; mp; 150 – 152 °C; IR 2933 (NH), 1770 (C=O), 1734 (C=O), 1624 (C=O), 1508 (C=C); ^1^H NMR δ (500 MHz, DMSO-*d_6_*) 9.20 (1H, d, *J* = 8.5 Hz, N*H*), 8.08 (2H, dd, *J* = 7.8, 1.6 Hz, Ar*H*), 7.64 – 7.57 (3H, m, Ar*H*), 7.47 (1H, d, *J* = 1.6 Hz, Ar*H*), 7.33 – 7.28 (5H, m, Ar*H*), 7.26 – 7.22 (3H, m, Ar*H*), 6.99 (1H, s, =C*H*), 6.85 (2H, d, *J* = 8.7 Hz, Ar*H*), 5.43 (1H, dd, *J* = 8.5, 4.2 Hz, NHC*H*), 5.33 (1H, d, *J* = 4.2 Hz, SC*H*N), 5.15 (1H, d, *J* = 11.7 Hz, OC*H_A_*H_B_Ar), 5.07 (1H, d, *J* = 11.7 Hz, OCH_A_*H_B_*Ar), 4.92 (1H, *J* = 12.2 Hz, ArOC*H_C_*H_D_), 4.72 (1H, s, NC*H*COO), 4.46 (1H, d, *J* = 12.2 Hz, ArOCH_C_*H_D_*), 3.72 (3H, s, C*H_3_*), 3.58 (1H, d, *J* = 14.1 Hz, COC*H_E_*H_F_), 3.56 (1H, d, *J* = 13.8 Hz, SC*H_G_*H_H_), 3.53 (1H, d, *J* = 14.1 Hz, COCH_E_*H_F_*), 3.02 (1H, d, *J* = 13.8 Hz, SCH_G_*H_H_*); ^13^C NMR δ (126 MHz, DMSO-*d_6_*) 176.53 (C), 171.06 (C), 166.74 (C), 165.57 (C), 162.83 (C), 159.80 (C), 151.54 (C), 147.47 (C), 139.79 (C), 136.10 (C), 132.20 (CH), 131.65 (C), 130.94 (2 × CH), 129.59 (2 × CH), 129.47 (2 × CH), 128.75 (2 × CH), 127.09 (C), 127.02 (CH), 126.68 (2 × CH), 119.13 (C), 114.15 (2 × CH), 111.99 (CH), 106.58 (CH), 106.45 (CH), 71.30 (C), 67.58 (CH_2_), 66.36 (CH_2_), 59.58 (CH), 55.67 (CH), 55.44 (CH_3_), 52.75 (CH), 42.33 (CH_2_), 26.84 (CH_2_); *m/z* (ESI+, MeCN) 705 ([M+H]^+^,100%); HRMS (ESI+, MeCN) [M+H]^+^ found 705.1925, C_39_H_33_N_2_O_9_S requires 705.1901. Minor diastereomer: R_t_ (method 1) = 15.74 min; ^1^H NMR δ (500 MHz, DMSO-*d_6_*) diagnostic peaks: 9.13 (1H, d, *J* = 8.5 Hz, N*H*), 5.75 (1H, dd, *J* = 8.4, 4.8 Hz, NHC*H*), 5.15 (1H, d, *J* = 4.2 Hz, SC*H*N), 4.71 (1H, s, NC*H*COO), 3.73 (3H, s, C*H_3_*).

(4-Methoxyphenyl)methyl (2′*S*,2*R,*6*R*,7*R*)-10′-hydroxy-8,9′-dioxo-7′-phenyl-7-(2-phenylacetamido)-3′,8′-dihydro-5-thia-1-azaspiro[1′,4′-dioxino-3,2′-bicyclo[4.2.0]octane]-2-carboxylate (**17**): 40.3 mg (51%); R_t_ (method 1) = 15.27 min; R_f_ (DCM:EtOAc, 8:1) = 0.38; mp 113 – 115 °C; IR 2954 (NH), 1747 (C=O), 1734 (C=O), 1653 (C=O), 1516 (C=C); ^1^H NMR δ (500 MHz, DMSO-*d_6_*) 12.91 (1H, s, O*H*), 9.20 (1H, d, *J* = 8.5 Hz, N*H*), 8.11 (2H, d, *J* = 7.1 Hz, Ar*H*), 7.66 – 7.59 (3H, m, Ar*H*), 7.34 – 7.28 (4H, m, Ar*H*), 7.25 (1H, m, Ar*H*), 7.15 (2H, d, *J* = 8.6 Hz, Ar*H*), 7.07 (1H, s, =C*H*), 6.94 (1H, s, Ar*H*), 6.74 (2H, d, *J* = 8.6 Hz, Ar*H*), 5.43 (1H, d, *J* = 8.5, 4.2 Hz, NHC*H*), 5.34 (1H, d, *J* = 4.2 Hz, SC*H*N), 5.11 (1H, d, *J* = 11.8 Hz, OC*H_A_*H_B_Ar), 5.03 (1H, d, *J* = 12.2 Hz, ArOC*H*_C_H_D_), 4.93 (1H, d, *J* = 11.8 Hz, OCH_A_*H_B_*Ar), 4.69 (1H, s, N*H*COO), 4.34 (1H, d, *J* = 12.2 Hz, ArOCH_C_*H_D_*), 3.65 – 3.63 (4H, m, SC*H_E_*H_F_, C*H*_3_), 3.58 (1H, d, *J* = 14.1 Hz, COC*H_G_*H_H_), 3.53 (1H, d, *J* = 14.1 Hz, COCH_G_*H_H_*), 3.07 (1H, d, *J* = 13.8 Hz, SCH_E_*H_F_*); ^13^C NMR δ (126 MHz, DMSO-*d_6_*) 183.04 (C), 171.05 (C), 166.81 (C), 165.64 (C), 164.25 (C), 159.66 (C), 150.46 (C), 148.87 (C), 148.36 (C), 136.10 (C), 132.73 (CH), 131.08 (C), 130.39 (2 × CH), 129.67 (2 × CH), 129.47 (2 × CH), 128.75 (2 × CH), 127.02 (CH), 126.94 (2 × CH), 126.85 (C), 126.05 (C), 114.03 (2 × CH), 106.12 (C), 105.00 (CH), 95.61 (CH), 70.44 (C), 67.73 (CH_2_), 66.78 (CH_2_), 59.53 (CH), 55.79 (CH), 55.38 (CH_3_), 52.32 (CH), 42.34 (CH_2_), 27.11 (CH_2_); *m/z* (ESI+, MeCN) 721 ([M+H]^+^, 100%); HRMS (ESI+, MeCN) [M+H]^+^ found 721.1846, C_39_H_33_N_2_O_10_S requires 721.1850.

(4-Methoxyphenyl)methyl (2′*S*,2*R*,6*R*,7*R*)-14’,15’-dihydroxy-8,10’,17’-trioxo-7-(2-phenylacetamido)-3’,6’,11’,18’-tetraoxa-5-thia-1-azaspiro-bicyclo[4.2.0]octane-3,4’-pentacyclo-icosane-1’,2’,8’,12’,13’,15’-hexaene-2-carboxylate (**19**): 17.5 mg (28%); R_t_ (method 2) = 27.61 min; mp 131 – 133 °C; IR 2978 (NH), 1734 (C=O), 1716 (C=O), 1541 (C=O); ^1^H NMR δ (500 MHz, DMSO-*d_6_*) 9.23 (1H, d, *J* = 8.5 Hz, N*H*), 7.47 (1H, s, Ar*H*), 7.35 – 7.21 (8H, m, Ar*H*), 6.84 (2H, d, *J* = 8.5 Hz, Ar*H*), 5.54 (1H, dd, *J* = 8.5, 4.2 Hz, NHC*H*), 5.34 (1H, d, *J* = 4.2 Hz, SC*H*N), 5.20 (1H, d, *J* = 11.8 Hz, OC*H_A_*H_B_Ar), 5.06 (1H, d, *J* = 11.8 Hz, OCH_A_*H_B_*Ar), 5.01 (1H, d, *J* = 12.1 Hz, ArO*H_C_*H_D_), 4.77 (1H, s, NC*H*CO), 4.59 (1H, d, *J* = 12.1 Hz, ArOH_C_*H_D_*), 3.73 (3H, s, C*H*_3_), 3.60 – 3.51 (3H, m, SC*H_E_*H_F_, COC*H_2_*), 3.07 (1H, d, *J* = 13.8 Hz, SCH_E_*H_F_*); ^13^C NMR δ (126 MHz, DMSO-*d*_6_) 171.11 (C), 166.67 (C), 165.64 (C), 159.82 (2 × C), 158.91 (C), 158.86 (C), 149.42 (C), 142.78 (C), 137.22 (C), 136.85 (C), 136.10 (2 × C), 135.55 (C), 130.94 (2 × CH), 129.49 (2 × CH), 128.77 (2 × CH), 127.17 (C), 127.04 (CH), 114.11 (2 × CH), 113.76 (C), 113.28 (CH), 112.00 (C), 111.24 (C), 110.83 (CH), 71.79 (C), 67.54 (CH_2_), 66.48 (CH_2_), 59.65 (CH), 55.72 (CH), 55.42 (CH_3_), 52.76 (CH), 42.35 (CH_2_), 26.74 (CH_2_); *m/z* (ESI-, MeCN) 751 ([M-H]^-^, 100%); HRMS (ESI-, MeCN) [M-H]^-^ found 751.1264, C_38_H_27_N_2_O_13_S requires 751.1228. Minor diastereomer: R_t_ (method 2) = 27.92 min; ^1^H NMR δ (500 MHz, DMSO-*d_6_*) diagnostic peaks: 9.24 (1H, d, *J* = 7.9 Hz, N*H*), 5.42 (1H, dd, *J* = 7.9, 4.3 Hz, NHC*H*), 5.21 (1H, d, *J* = 4.3 Hz, SC*H*N), 4.74 (1H, s, NC*H*CO), 3.77 (3H, s, C*H_3_*).

### 3.4. Typical Procedure for PMB Deprotection 

Anisole (0.15 mL, 1.34 mmol) was added to a stirred solution of spiro-cephalosporin **5** (30.0 mg, 0.05 mmol) in DCM (4 mL) and was cooled to 0 °C. TFA (0.21 mL, 2.68 mmol) was added dropwise to the reaction mixture and the mixture was stirred at 0 °C for 1 h, then at rt for 3 h. The reaction mixture was concentrated in vacuo and the crude product was purified by column chromatography (DCM:MeOH:AcOH, 96:4:1) to give the desired product **20** [45].

### 3.5. Characterisation of PMB Deprotected Products 

(2′*S*,2*R*,6*R*,7*R*)-8-Oxo-7-(2-phenylacetamido)-5-thia-1-azaspiro[1′,4′-benzodioxine-3,2’-bicyclo[4.2.0]octane]-2-carboxylic acid (**20**): 16.6 mg (71%); R_t_ (method 1) = 12.90 min; R_f_ (DCM:MeOH:AcOH, 96:4:1) = 0.25; mp 206 – 208 °C; IR (neat, cm^-1^) 3400 – 3061 (OH), 2887 (NH), 1747 (C=O), 1625 (C=O); ^1^H NMR δ (500 MHz, DMSO-*d_6_*) 9.18 (1H, d, *J* = 8.5 Hz, N*H*), 7.35 – 7.26 (4H, m, Ar*H*), 7.26 – 7.22 (1H, m, Ar*H*), 7.01 – 6.95 (1H, m, Ar*H*), 6.91 – 6.88 (2H, m, Ar*H*), 6.86 – 6.81 (1H, m, Ar*H*), 5.39 (1H, dd, *J* = 8.5, 4.2 Hz, NHC*H*), 5.29 (1H, d, *J* = 4.2 Hz, SC*H*N), 4.73 (1H, d, *J* = 12.0 Hz, ArO*H_A_*H_B_), 4.51 (1H, s, NC*H*COO), 4.20 (1H, d, *J* = 12.0 Hz, ArOH_A_*H_B_*), 3.65 (1H, d, *J* = 13.6 Hz, SC*H_C_*H_D_), 3.57 (1H, d, *J* = 14.0 Hz, COC*H_E_*H_F_), 3.52 (1H, d, *J* = 14.0 Hz, COCH_E_*H_F_*), 2.86 (1H, d, *J* = 13.6 Hz, SCH_C_*H_D_*); ^13^C NMR δ (126 MHz, DMSO-*d*_6_) 171.03 (C), 168.41 (C), 165.48 (C), 142.36 (C), 141.79 (C), 136.20 (C), 129.49 (2 × CH), 128.73 (2 × CH), 126.99 (CH), 122.76 (CH), 122.18 (CH), 117.87 (CH), 117.66 (CH), 70.84 (C), 66.12 (CH_2_), 59.22 (CH), 55.40 (CH), 53.40 (CH), 42.28 (CH_2_), 26.96 (CH_2_); *m/z* (ESI+, MeCN) 463 ([M+Na]^+^, 29%), 458 ([M+NH_4_]^+^, 15), 441 ([M+H]^+^, 100); HRMS (ESI+, MeCN) [M+H]^+^ found 441.1104, C_22_H_21_N_2_O_6_S requires 441.1115.

(2′*S*,2*R*,6*R*,7*R*)-7′,8-Dioxo-7-(2-phenylacetamido)-3′,7′-dihydro-5-thia-1-azaspiro[1′,4′-dioxino-3,2′-bicyclo[4.2.0]octane]-2-carboxylic acid (**21**): 37.0 mg (93%); R_t_ (method 1) = 12.03 min; R_f_ (DCM:MeOH:AcOH, 92:8:1) = 0.40; mp 210 – 212 °C; IR 3304 (NH), 1718 (C=O), 1560 (C=O), 1500 (C=C); ^1^H NMR δ (500 MHz, DMSO-*d*_6_) 9.15 (1H, d, *J* = 8.5 Hz, N*H*), 7.94 (1H, d, *J* = 9.6 Hz, COC*H*=CH), 7.35 – 7.27 (4H, m, Ar*H*), 7.26 – 7.20 (2H, m, Ar*H*), 7.08 (1H, s, Ar*H*), 6.34 (1H, d, *J* = 9.6 Hz, COCH=C*H*), 5.34 (2H, m, NHC*H*, SC*H*N), 4.78 (1H, d, *J* = 12.0 Hz, OC*H_A_*H_B_), 4.40 (1H, br s, NC*H*COO), 4.38 (1H, d, *J* = 12.0 Hz, OCH_A_*H_B_*), 3.76 (1H, br d, *J* = 13.6 Hz, SC*H_C_*H_D_), 3.57 (1H d, *J* = 14.1 Hz, COC*H_E_*H_F_), 3.52 (1H d, *J* = 14.1 Hz, COCH_E_*H_F_*), 2.82 (1H, br d, *J* = 13.6 Hz, SCH_C_*H_D_*); ^13^C NMR δ (126 MHz, DMSO-*d_6_*) 171.02 (C), 168.00 (C), 165.24 (C), 160.63 (C), 149.22 (C), 145.81 (C), 144.29 (CH), 138.84 (C), 136.23 (C), 129.49 (2 × CH), 128.73 (2 × CH), 126.98 (CH), 115.93 (CH), 114.44 (CH), 113.99 (C), 104.94 (CH), 71.11 (C), 66.75 (CH_2_), 59.10 (CH), 55.43 (CH), 54.44 (CH), 42.29 (CH_2_), 26.94 (CH_2_); *m/z* (ESI+, MeCN) 531 ([M+Na]^+^, 100%), 509 ([M+H]^+^, 33); HRMS (ESI+, MeCN) [M+H]^+^ found 509.1038, C_25_H_21_N_2_O_8_S requires 509.1013. 

(2′*S*,2*R*,6*R*,7*R*)-7′,8-Dioxo-7-(2-phenylacetamido)-3′,7′,8′,9′,10′,11′-hexahydro-1′,4′,6′-trioxa-5-thia-1-azaspiro[bicyclo[4.2.0]octane-3,2’-tetraphene]-2-carboxylic acid (**22**): 60.4 mg (74%); R_t_ (method 1) = 12.94 min; R_f_ (DCM:MeOH:AcOH, 95:5:1) = 0.50; mp 218 – 220 °C; IR 3400 – 3200 (OH), 2934 (NH), 1751 (C=O), 1676 (C=O); ^1^H NMR δ (500 MHz, DMSO-*d_6_*) 9.19 (1H, *J* = 8.5 Hz, N*H*), 7.32 – 7.25 (5H, m, Ar*H*), 7.13 (1H, s, Ar*H*), 7.08 (1H, s, Ar*H*), 5.40 (1H, dd, *J* = 8.5, 4.1 Hz, NHC*H*), 5.30 (1H, d, *J* = 4.1 Hz, SC*H*N), 4.81 (1H, d, *J* = 12.1 Hz, ArOC*H_A_*H_B_), 4.54 (1H, s, NC*H*COO), 4.36 (1H, d, *J* = 12.1 Hz, ArOCH_A_*H_B_*), 3.66 (1H, d, *J* = 13.6 Hz, SC*H_C_*H_D_), 3.57 (1H, d, *J* = 14.1 Hz, COC*H_E_*H_F_), 3.52 (1H, d, *J* = 14.1 Hz, COCH_E_*H_F_*), 2.92 (1H, d, *J* = 13.6 Hz, SCH_C_*H_D_*), 2.76 – 2.64 (2H, m, C_11′_*H*), 2.42 -2.39 (2H, m, C_11′_*H*), 1.77 – 1.69 (4H, m, C_9′_*H_2_*C_10′_*H_2_*). ^13^C NMR δ (126 MHz, DMSO-*d_6_*) 171.03 (C), 168.27 (C), 165.53 (C), 161.18 (C), 147.36 (C), 147.01 (C), 144.36 (C), 138.52 (C), 136.18 (C), 129.49 (2 × CH), 128.74 (2 × CH), 127.00 (CH), 121.48 (C), 115.10 (C), 111.65 (CH), 104.85 (CH), 71.05 (C), 66.52 (CH_2_), 59.31 (CH), 55.48 (CH), 53.37 (CH), 42.31 (CH_2_), 26.90 (CH_2_), 25.18 (CH_2_), 24.13 (CH_2_), 21.60 (CH_2_), 21.31 (CH_2_). *m/z* (ESI+, MeCN) 585 ([M+Na]^+^, 23%), 580 ([M+NH_4_]^+^, 35), 563 ([M+H]^+^, 100); HRMS (ESI+, MeCN) [M+H]^+^ found 563.1476, C_29_H_27_N_2_O_8_S requires 563.1483.

(2′*S*,2*R*,6*R*,7*R*)-7′,8-Dioxo-9′-phenyl-7-(2-phenylacetamido)-3′,7′dihydro-5-thia-1-azaspiro[1′,4′-dioxino-3,2′-bicyclo[4.2.0]octane]-2-carboxylic acid (**23**): 150 mg (74%); R_t_ (method 1) = 13.35 min; R_f_ (DCM:MeOH:AcOH, 90:10:1) = 0.38; mp 182 – 184 °C; IR 3300 – 2954 (OH), 2924 (NH), 1724 (C=O), 1626 (C=O), 1506 (C=C); ^1^H NMR δ (500 MHz, DMSO-*d_6_*) 13.52 (1H, s, O*H*), 9.18 (1H, d, *J* = 8.5 Hz, N*H*), 7.59 (3H, m, Ar*H*), 7.51 (2H, m, Ar*H*), 7.33 – 7.27 (4H, m, Ar*H*), 7.25 – 7.22 (2H, m, Ar*H*), 6.75 (1H, s, Ar*H*), 6.32 (1H, s, =C*H*), 5.41 (1H, dd, *J* = 8.5, 4.2 Hz, NHC*H*), 5.26 (1H, d, *J* = 4.2 Hz, SC*H*N), 4.88 (1H, d, *J* = 12.2 Hz, ArOC*H_A_*H_B_), 4.54 (1H, s, NC*H*COO), 4.39 (1H, d, *J* = 12.2 Hz, ArOCH_A_*H_B_*), 3.57 (1H, d, *J* = 14.1 Hz, COC*H_E_*H_F_), 3.55 (1H, d, *J* = 13.8 Hz, SC*H_C_*H_D_), 3.52 (1H, d, *J* = 14.1 Hz, COCH_E_*H_F_*), 2.93 (1H, d, *J* = 13.8 Hz, SCH_C_*H_D_*); ^13^C NMR δ (126 MHz, DMSO-*d_6_*) 171.02 (C), 168.26 (C), 165.63 (C), 160.27 (C), 154.80 (C), 149.44 (C), 145.79 (C), 138.39 (C), 136.15 (C), 135.24 (C), 130.27 (CH), 129.49 (2 × CH), 129.46 (2 × CH), 128.79 (2 × CH), 128.74 (2 × CH), 127.01 (CH), 114.17 (CH), 113.74 (C), 113.43 (CH), 105.75 (CH), 70.85 (C), 66.63 (CH_2_), 59.39 (CH), 55.53 (CH), 52.88 (CH), 42.32 (CH_2_), 26.93 (CH_2_); *m/z* (ESI+, MeCN) 585 ([M+H]^+^, 100); HRMS (ESI+, MeCN) [M+H]^+^ found 585.1315, C_31_H_25_N_2_O_8_S requires 585.1326. 

(2′*S*,2*R*,6*R*,7*R*)-8,9′-Dioxo-7′-phenyl-7-(2-phenylacetamido)-3′,9′-dihydro-5-thia-1-azaspiro[1′,4′-dioxino -3,2′-bicyclo[4.2.0]octane]-2-carboxylic acid (**24**): 61.9 mg (97%); R_t_ (method 1) = 13.47 min; R_f_ (DCM:MeOH:AcOH, 90:10:1) = 0.42; mp 177 – 178 °C; IR 3430 – 3120 (OH), 2924 (NH), 1751 (C=O), 1622 (C=O); ^1^H NMR δ (500 MHz, DMSO-*d_6_*) 13.63 (1H, s, O*H*), 9.18 (1H, d, *J* = 8.5 Hz, N*H*), 8.09 (2H, dd, *J* = 8.0, 1.6 Hz, Ar*H*), 7.61 (3H, m, Ar*H*), 7.52 (1H, s, Ar*H*), 7.36 (1H, s, Ar*H*), 7.34 – 7.27 (4H, m, Ar*H*), 7.25 (1H, m, Ar*H*), 6.96 (1H, s, =C*H*), 5.42 (1H, dd, *J* = 8.5, 4.2 Hz, NHC*H*), 5.31 (1H, d, *J* = 4.2 Hz, SC*H*N), 4.96 (1H, d, *J* = 12.2 Hz, ArOC*H_A_*H_B_), 4.59 (1H, s, NC*H*COO), 4.43 (1H, d, *J* = 12.2 Hz, ArOCH_A_*H_B_*), 3.65 (1H, d, *J* = 13.7 Hz, SC*H_C_*H_D_), 3.58 (1H, d, *J* = 14.1 Hz, COC*H_E_*H_F_Ar), 3.53 (1H, d, *J* = 14.1 Hz, COCH_E_*H_F_*Ar), 3.00 (1H, d, *J* = 13.7 Hz, SCH_C_*H_D_*); ^13^C NMR δ (126 MHz, DMSO-*d_6_*) 176.56 (C), 171.04 (C), 168.27 (C), 165.60 (C), 162.95 (C), 151.60 (C), 147.71 (C), 140.14 (C), 136.15 (C), 132.21 (CH), 131.70 (C), 129.59 (2 × CH), 129.50 (2 × CH), 128.75 (2 × CH), 127.02 (CH), 126.74 (2 × CH), 119.10 (C), 111.64 (CH), 106.64 (CH), 106.46 (CH), 71.13 (C), 66.60 (CH_2_), 59.45 (CH), 55.51 (CH), 52.94 (CH), 42.34 (CH_2_), 27.02 (CH_2_); *m/z* (ESI+, MeCN) 585 ([M+H]^+^,100%); HRMS (ESI+, MeCN) [M+H]^+^ found 585.1353, C_31_H_25_N_2_O_8_S requires 585.1326. 

(2′*S*,2*R*,6*R*,7*R*)-10′-Hydroxy-8,9′-dioxo-7′-phenyl-7-(2-phenylacetamido)-3′,9′-dihydro-5-thia-1-azaspiro[1′,4′-dioxino-3,2′-bicyclo[4.2.0]octane]-2-carboxylic acid (**25**): 60.0 mg (63%); R_t_ (method 1) = 13.73 min; R_f_ (DCM:MeOH:AcOH, 95:5:1) = 0.47; mp 171 – 173 °C; IR 3500 – 3400 (OH), 3000 (NH), 1757 (C=O), 1609 (C=O), 1500 (C=C); ^1^H NMR δ (500 MHz, DMSO-*d_6_*) 13.61 (1H, brs, COO*H*), 12.94 (1H, s, ArO*H*), 9.18 (1H, d, *J* = 8.5 Hz, N*H*), 8.11 (2H, d, *J* = 7.0 Hz, Ar*H*), 7.66 – 7.57 (3H, m, Ar*H*), 7.34 – 7.28 (4H, m, Ar*H*), 7.25 (1H, m, Ar*H*), 7.03 (1H, s, =C*H*), 6.97 (1H, s, Ar*H*), 5.42 (1H, dd, *J* = 8.5, 4.1 Hz, NHC*H*), 5.31 (1H, d, *J* = 4.1 Hz, SC*H*N), 4.97 (1H, d, *J* = 12.1 Hz, ArOC*H_A_*H_B_), 4.56 (1H, s, N*H*COO), 4.40 (1H, d, *J* = 12.1 Hz, ArOCH_A_*H_B_*), 3.69 (1H, d, *J* = 13.7 Hz, SC*H_C_*H_D_), 3.58 (1H, d, *J* = 14.0 Hz, COC*H_E_*H_F_), 3.53 (1H, d, *J* = 14.0 Hz, COCH_E_*H_F_*), 2.98 (1H, d, *J* = 13.7 Hz, SCH_C_*H_D_*); ^13^C NMR δ (126 MHz, DMSO-*d_6_*) 183.15 (C), 171.02 (C), 168.18 (C), 165.56 (C), 164.36 (C), 150.51 (C), 149.03 (C), 148.68 (C), 136.14 (C), 132.70 (CH), 131.14 (C), 129.65 (2 × CH), 129.48 (2 × CH), 128.74 (2 × CH), 127.01 (3 × CH), 126.26 (C), 106.06 (C), 105.05 (CH), 95.64 (CH), 70.53 (C), 66.84 (CH_2_), 59.35 (CH), 55.55 (CH), 52.88 (CH), 42.33 (CH_2_), 26.98 (CH_2_); *m/z* (ESI-, MeCN) 599 ([M-H]^-^, 100%); HRMS (ESI-, MeCN) [M-H]^-^ found 599.1110, C_31_H_23_N_2_O_9_S requires 599.1119. 

(2′*S*,2*R*,6*R*,7*R*)-14’,15’-Dihydroxy-8,10’,17’-trioxo-7-(2-phenylacetamido)-3’,6’,11’,18’-tetraoxa-5-thia-1-azaspiro-bicyclo[4.2.0]octane-3,4’-pentacyclo-icosane-1’,2’,8’,12’,13’,15’-hexaene-2-carboxylic acid (**26**): 12.3 mg (77%); R_t_ (method 1) = 12.42 min; R_f_ (DCM:MeOH:AcOH, 86:14:1) = 0.23; mp 184 – 185 °C; IR 3486 – 3070 (OH), 2974 (NH), 1737 (C=O), 1614 (C=O); ^1^H NMR δ (600 MHz, DMSO-*d_6_*) 9.21 (1H, d= 8.5 Hz, N*H*), 7.46 (1H, s, Ar*H*), 7.42 (1H, s, Ar*H*), 7.34 – 7.30 (4H, m, Ar*H*), 7.26 – 7.25 (1H, m, Ar*H*), 5.42 (1H, dd, *J* = 8.5, 4.2 Hz, NHC*H*), 5.34 (1H, d, *J* = 4.1 Hz, SC*H*N), 5.06 (1H, d, *J* = 12.1 Hz, ArOC*H_A_*H_B_), 4.61 (1H, s, NC*H*COO), 4.54 (1H, d, *J* = 12.1 Hz, ArOCH_A_*H_B_*), 3.72 (1H, d, *J* = 13.5 Hz, SC*H_C_*H_D_), 3.59 (1H, d, *J* = 14.1 Hz, COC*H_E_*H_F_), 3.54 (1H, d, *J* = 14.1 Hz, COCH_E_*H_F_*), 3.03 (1H, d, *J* = 13.5 Hz, SCH_C_*H_D_*); ^13^C NMR δ (151 MHz, DMSO-*d_6_*) 171.06 (C), 168.18 (C), 165.56 (C), 159.02 (C), 158.88 (C), 149.41 (C), 143.19 (C), 137.30 (C), 136.81 (C), 136.17 (C), 135.78 (C), 132.07 (C), 129.51 (2 × CH), 128.76 (2 × CH), 127.02 (CH), 120.41 (C), 113.70 (C), 113.02 (CH), 112.01 (C), 111.16 (C), 110.86 (CH), 71.71 (C), 66.78 (CH_2_), 59.42 (CH), 55.53 (CH), 53.20 (CH), 42.36 (CH_2_), 26.97 (CH_2_); *m/z* (ESI-, MeCN) 631 ([M-H]^-^, 100%); HRMS (ESI-, MeCN) [M-H]^-^ found 631.0064, C_30_H_19_N_2_O_12_S requires 631.0653. 

## 4. Conclusions

Novel methodology for the synthesis of spiro-cephalosporins through a Michael-type reaction with the dihydrothiazine ring under mild basic conditions has been developed. A range of catechols were screened, and the spiro-cyclisation reactions were found to be highly diastereostereoselective (d.r. 14:1 to 8:1) with moderate to good yields (28-65%). The spirocyclisation products were transformed to more drug-like motifs through simple deprotection of the PMB-ester found in the cephalosporin precursor. Although the focus of this work has been the fusion of antioxidants, such as coumarins and flavonoids, with the cephalosporin core, we anticipate that spiro-cyclisation could be achieved with a range of other interesting catechol species, such as dopamine, apomorphine, catechin and caffeic acid. Given the recent rise in interest in the synthesis of spirocyclic motifs in medicinal chemistry [52], we anticipate that this new approach to spirocyclisation might be implemented across other target structures. 

## Figures and Tables

**Table 1:** Reaction scope for the synthesis of spiro-cephalosporins *^a^*.

Entry	Catechol	Major Product	Isolated Yield (%)	dr *^b^*
1	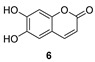	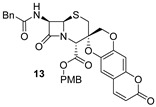	59	–*^c^*
2	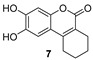	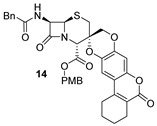	62	14:1
3	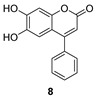	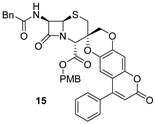	54	9:1
4	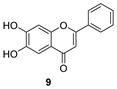	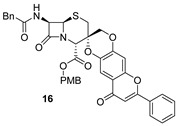	65	12:1
5	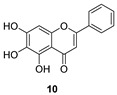	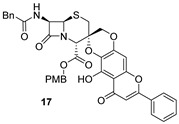	51	– *^c^*
6	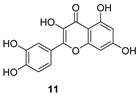	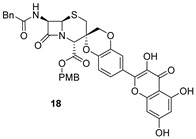	– *^d^*	– *^d^*
7	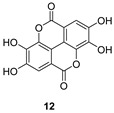	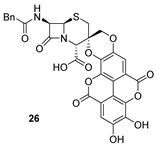	28	8:1

*^a^* Reagents and conditions: cephalosporin **4**, K_2_CO_3_, DMF, µW, 50 °C, 50 min. *^b^* dr values determined by high performance liquid chromatography (HPLC). *^c^* Only one diastereomer was found. *^d^* No product was isolated by column chromatography.

**Table 2:**
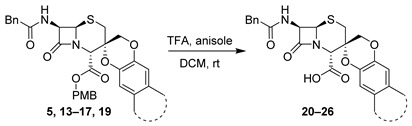
PMB deprotection of spiro-cephalosporins.

Entry	Starting Material	Product	Isolated Yield (%)
1	**5**	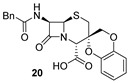	71
2	**13**	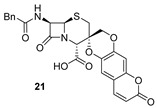	93
3	**14**	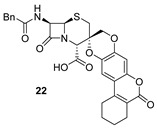	74
4	**15**	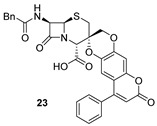	74
5	**16**	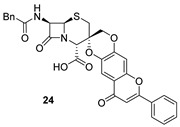	97
6	**17**	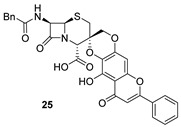	63
7	**19**	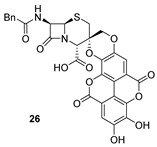	77

## Data Availability

Data is contained within the article or Supplementary Materials.

## Supplementary Materials

The following are available online at https://www.mdpi.com/article/10.3390/molecules26196035/s1, Figures S1–S14: ^1^H and ^13^C NMR spectra for compounds **5**, and **13**–**26**.

## Sample Availability

Samples of the compounds are not available from the authors.
