# Supplementary material for: New Methods for the Synthesis of Spirocyclic Cephalosporin Analogues
Source: Molecules. 2021 Oct 5;26(19):6035. doi: 10.3390/molecules26196035 (PMC8512572; doi:10.3390/molecules26196035)
Supplement: Supplementary file 1 [file molecules-26-06035-s001.zip › molecules-1369175-supplementary.pdf]

Supporting Information

New methods for the synthesis of spirocyclic cephalosporin analogues

Alan X. Zhao [1], Louise E. Horsfall [2] and Alison N. Hulme [1,*]

[1] EaStChem School of Chemistry, The University of Edinburgh, Joseph Black Building, David Brewster Road, Edinburgh EH9 3FJ, UK; s1117565@sms.ed.ac.uk (A.X.Z)
[2] Institute of Quantitative Biology, Biochemistry, and Biotechnology, School of Biological Science, The University of Edinburgh, Roger Land Building, Alexander Crum Brown Road, Edinburgh, EH9 3FF, UK; louise.horsfall@ed.ac.uk (L.E.H)
* Correspondence: Alison.Hulme@ed.ac.uk; Tel.: +44-131-650-4711

Page **Contents**

Page	Contents
S2	^{1}H and ^{13}C NMR spectra for spirocyclisation compounds **5** and **13-19**
S9	^{1}H and ^{13}C NMR spectra for PMB deprotection compounds **20-26**

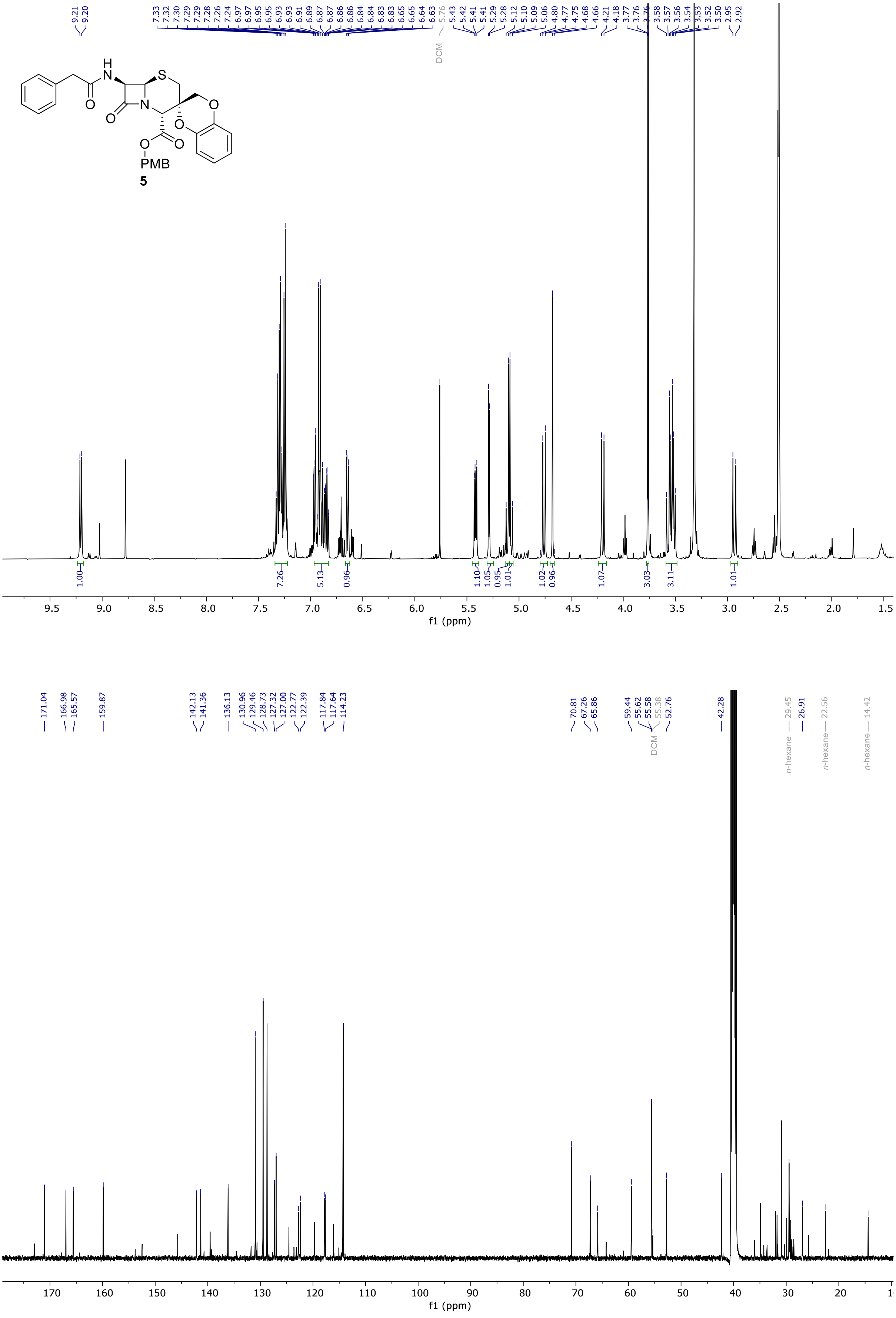

Figure S1. ^{1}H (top) and ^{13}C NMR (bottom) spectra of spiro-compound **5**.

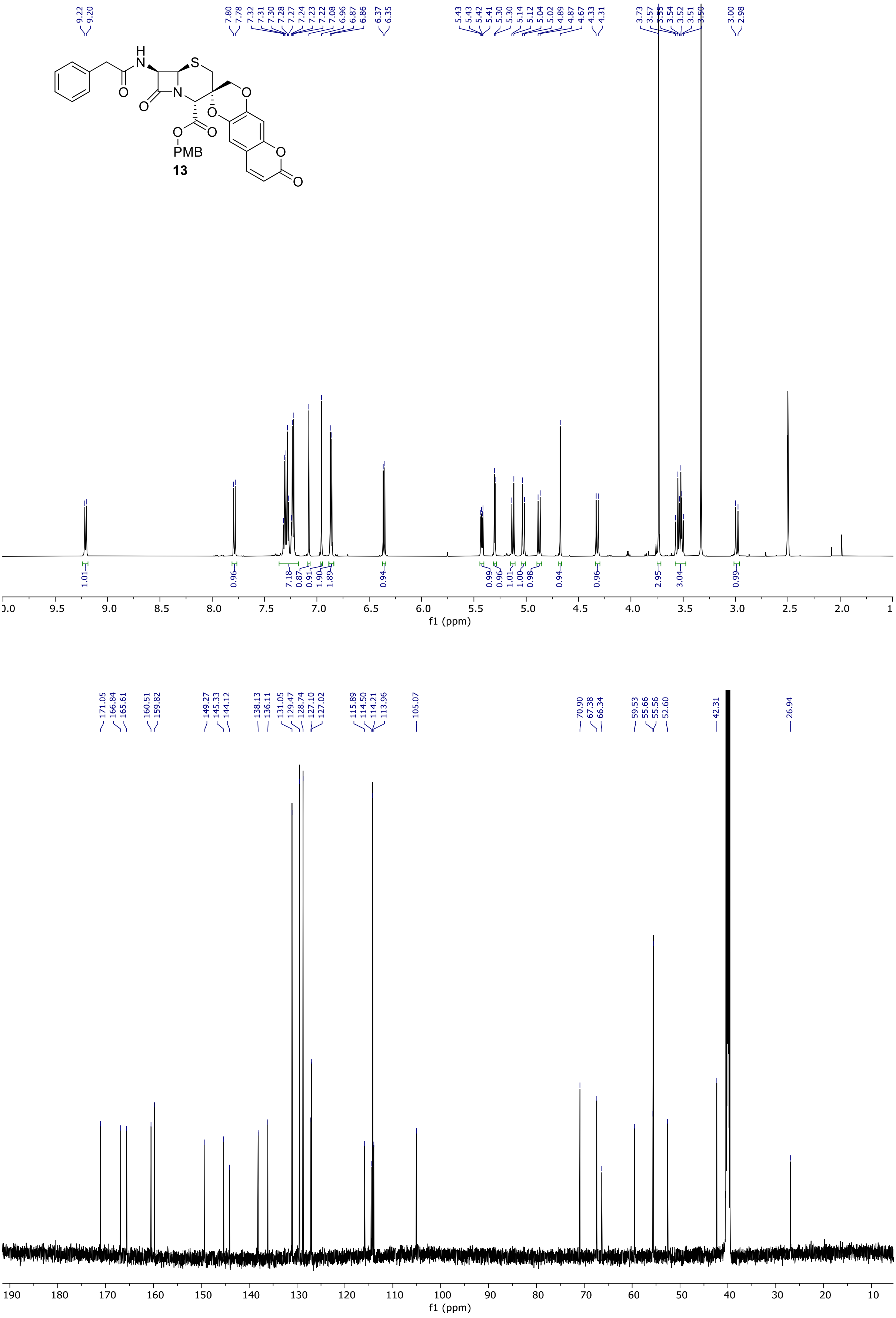

Figure S2. ^{1}H (top) and ^{13}C NMR (bottom) spectra of spiro-compound **13**.

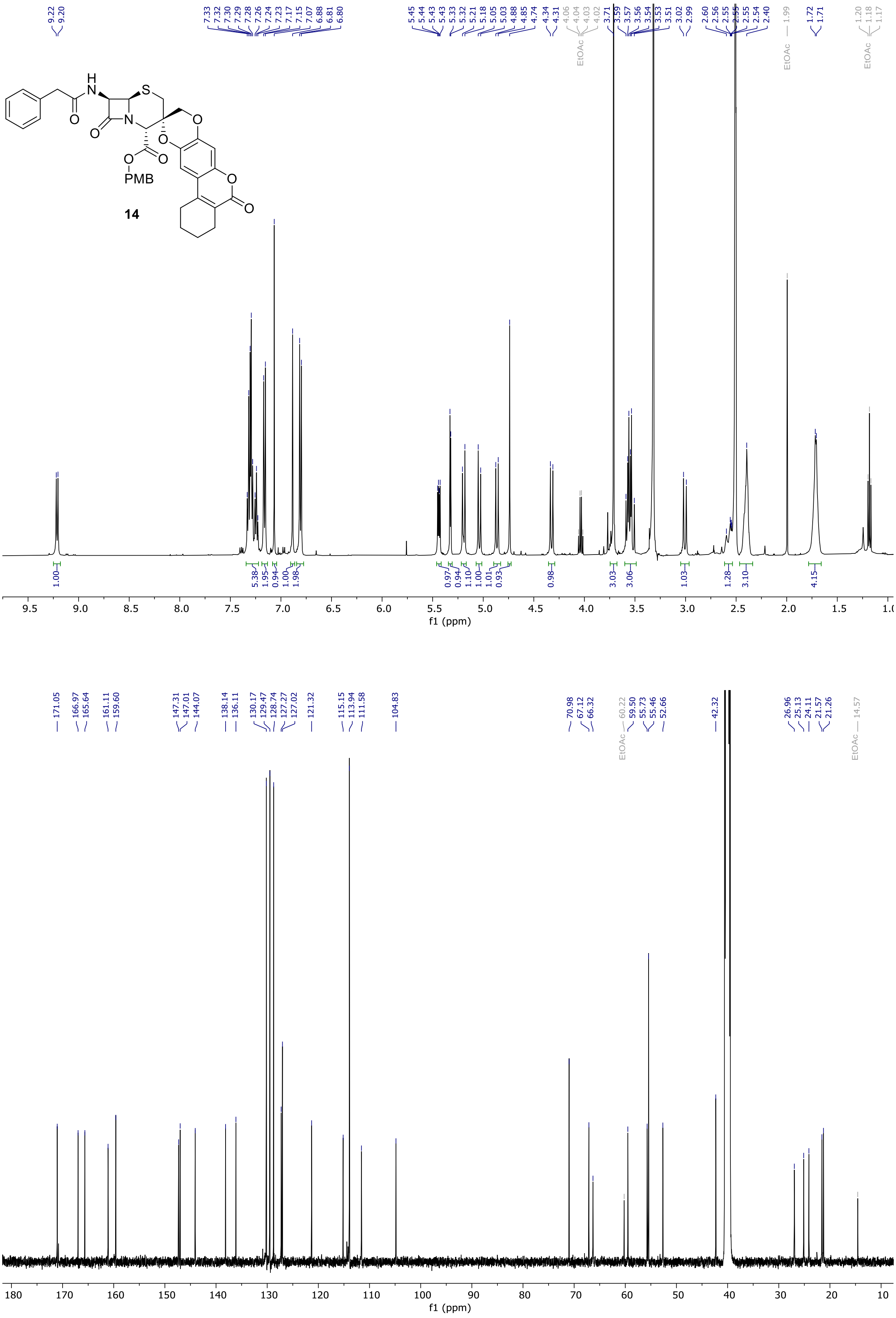

Figure S3. ^{1}H (top) and ^{13}C NMR (bottom) spectra of spiro-compound **14**.

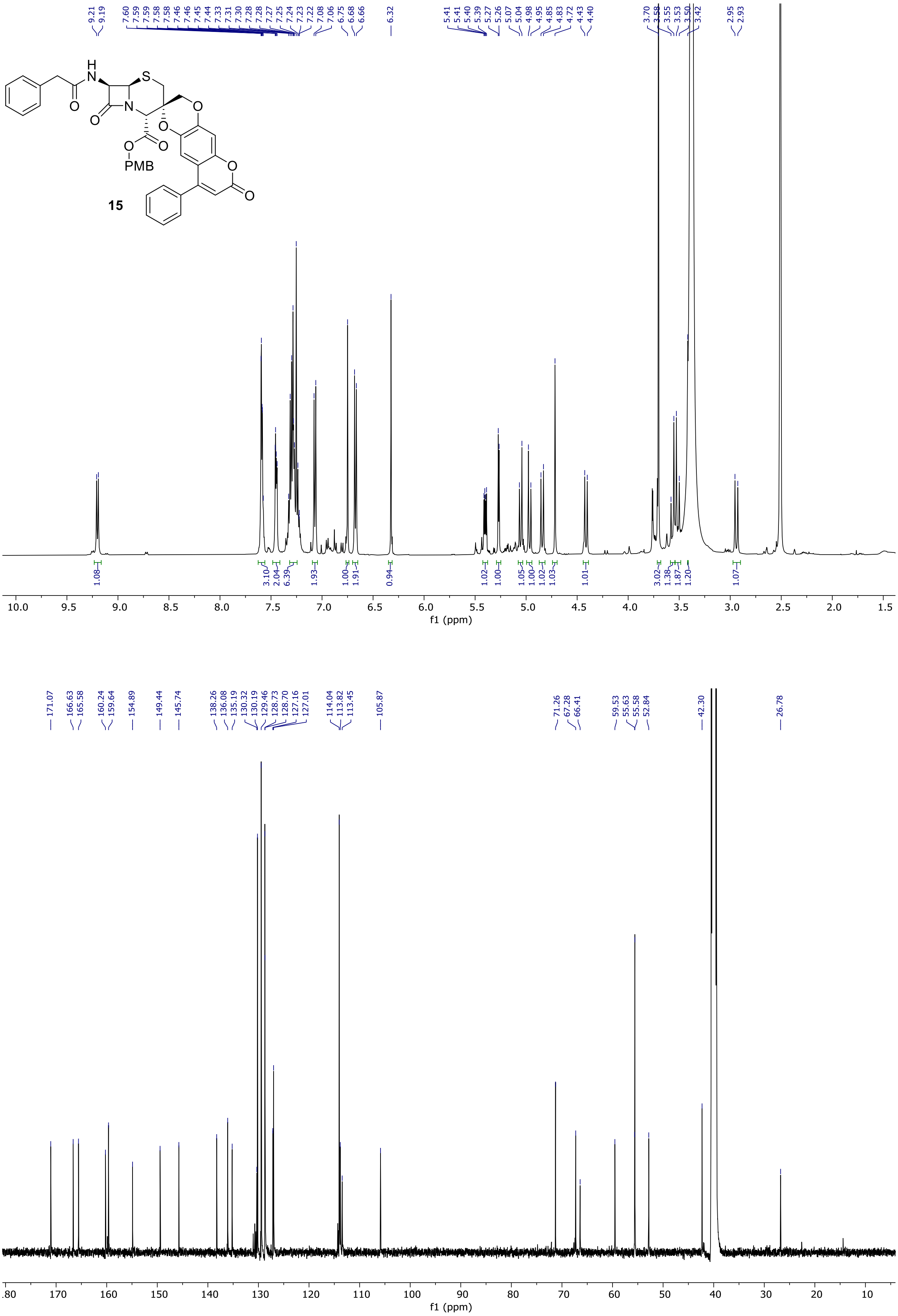

Figure S4. ^{1}H (top) and ^{13}C NMR (bottom) spectra of spiro-compound **15**.

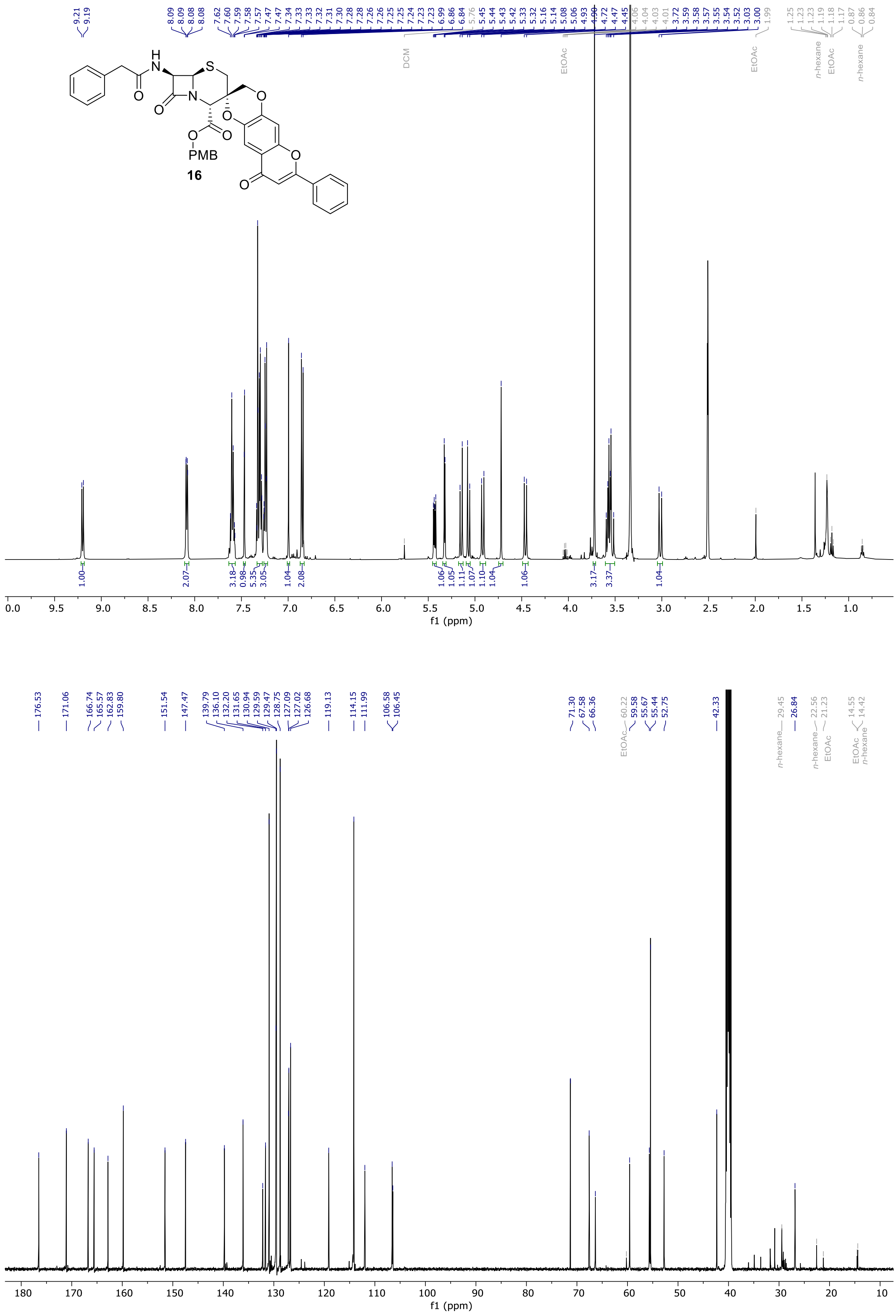

Figure S5. ^{1}H (top) and ^{13}C NMR (bottom) spectra of spiro-compound **16**.

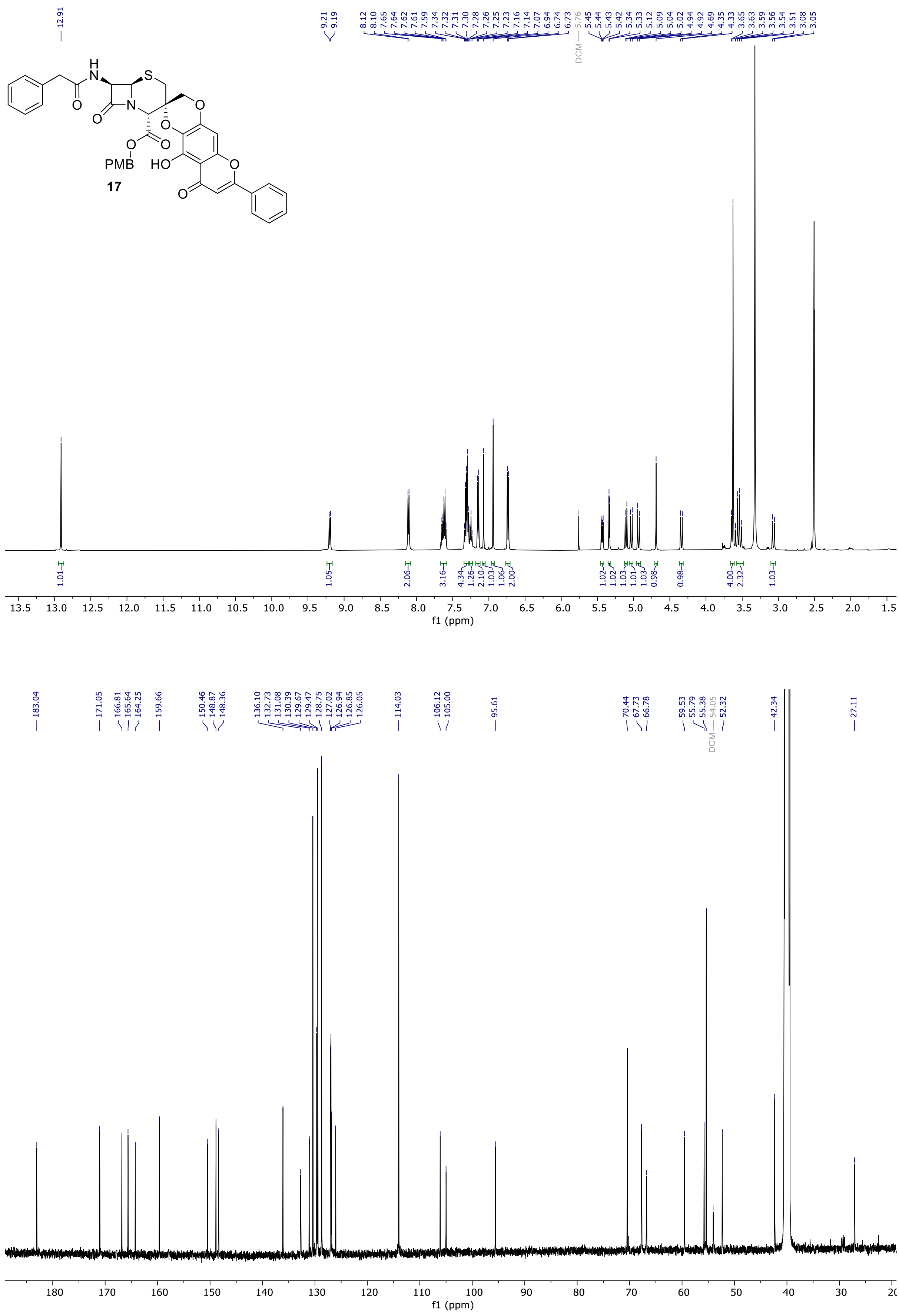

Figure S6. ^{1}H (top) and ^{13}C NMR (bottom) spectra of spiro-compound **17**.

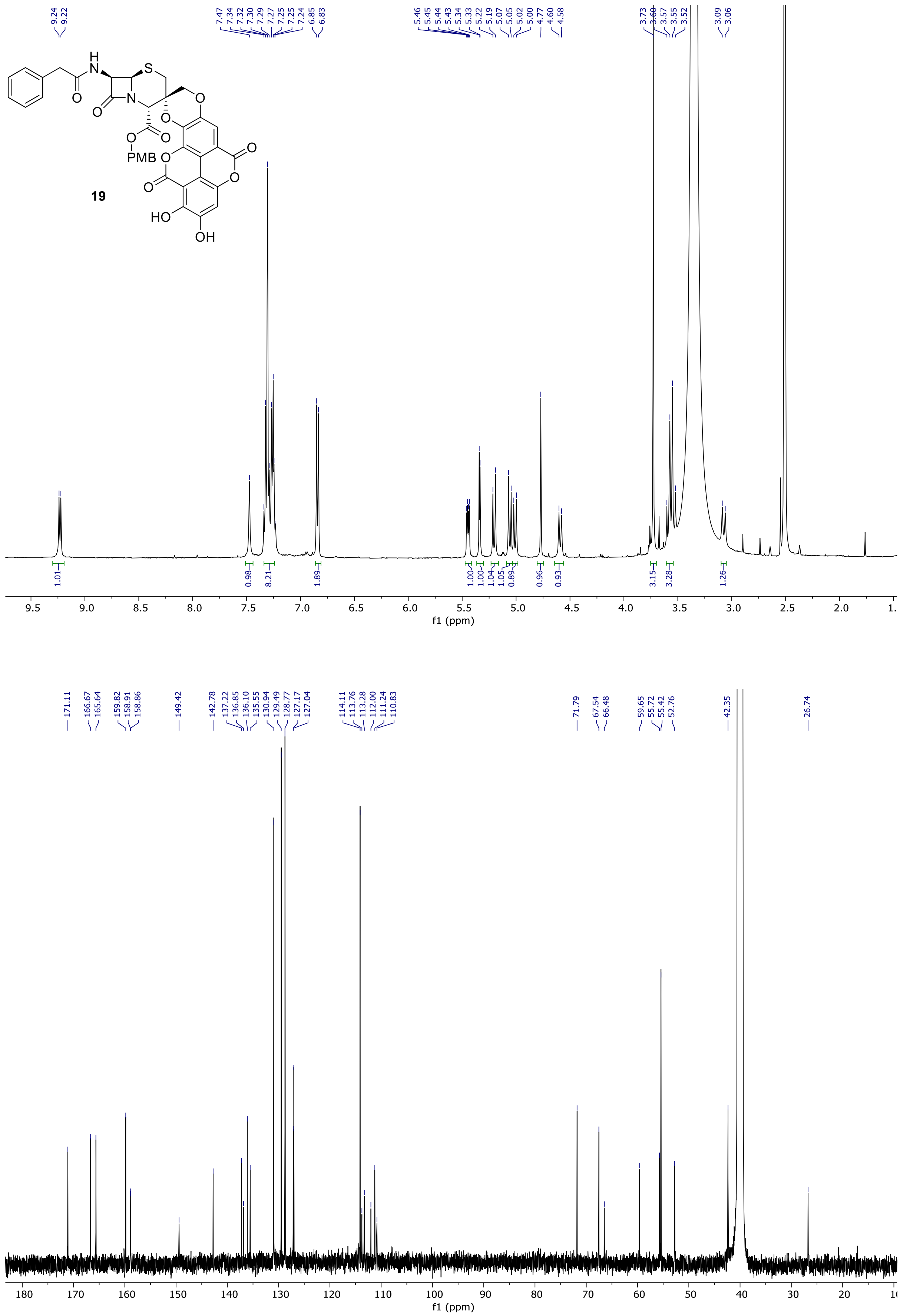

Figure S7. ^{1}H (top) and ^{13}C NMR (bottom) spectra of spiro-compound **19**.

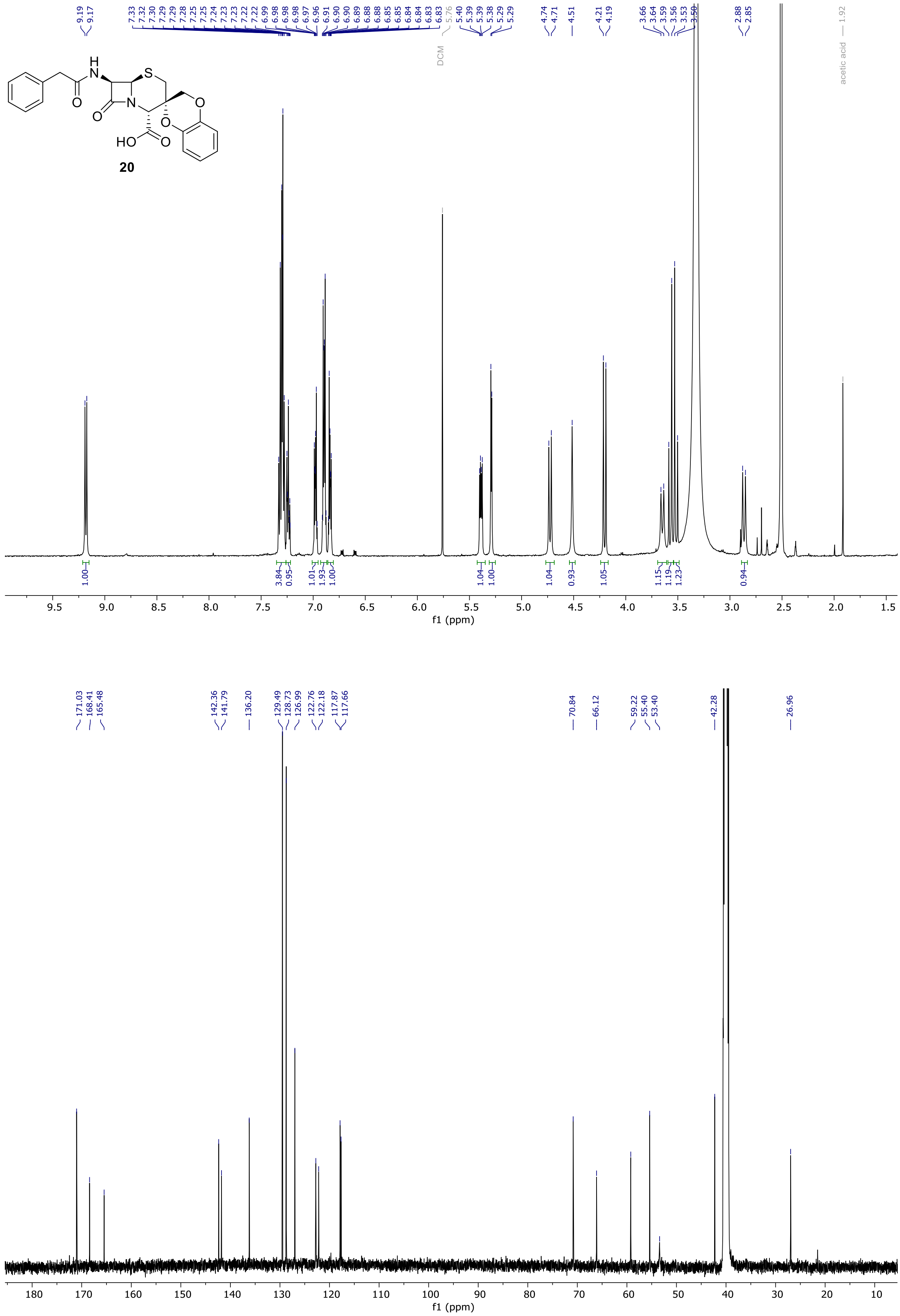

Figure S8. ^{1}H (top) and ^{13}C NMR (bottom) spectra of spiro-compound **20**.

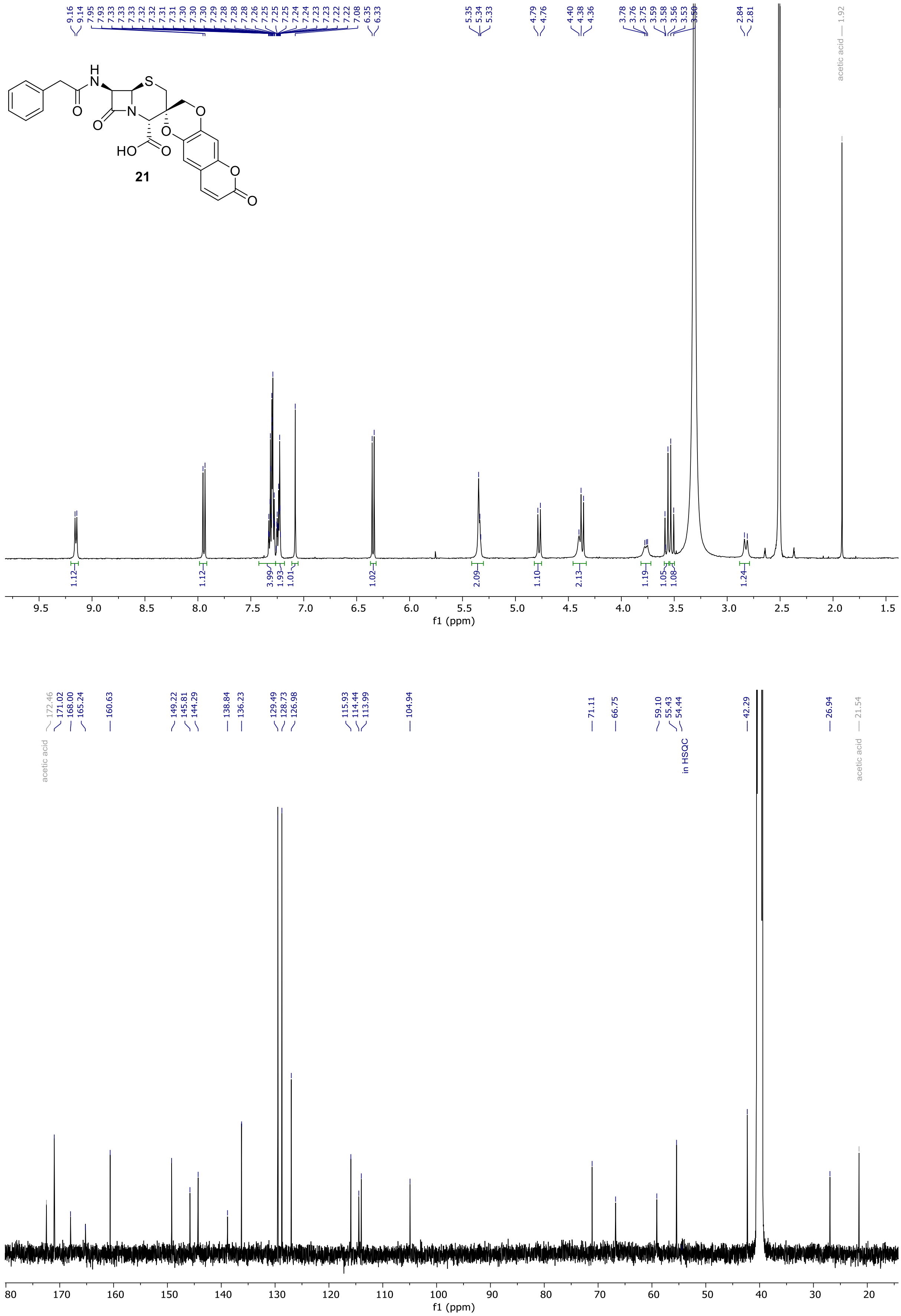

Figure S9. ^{1}H (top) and ^{13}C NMR (bottom) spectra of spiro-compound **21**.

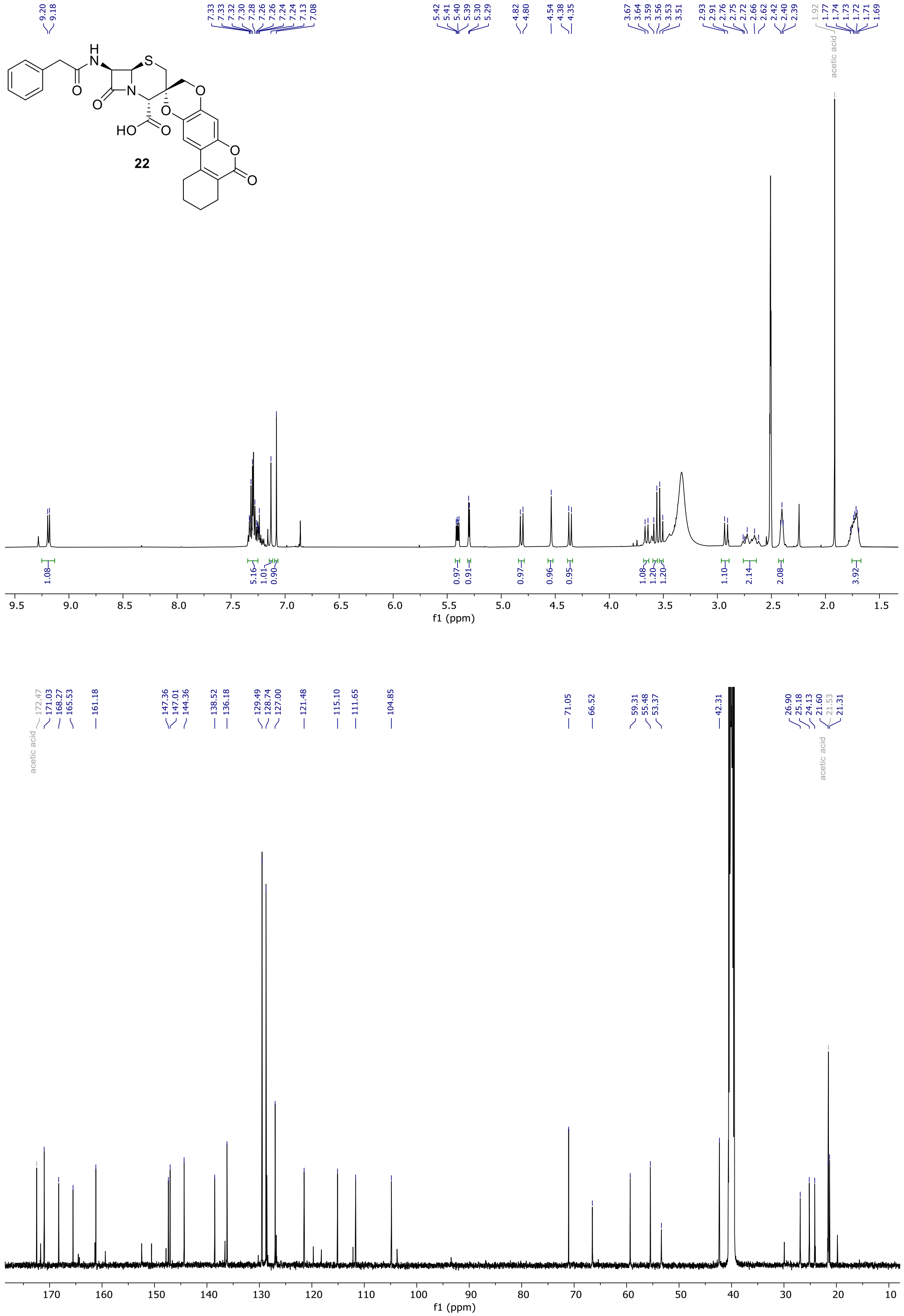

Figure S10. ^{1}H (top) and ^{13}C NMR (bottom) spectra of spiro-compound **22**.

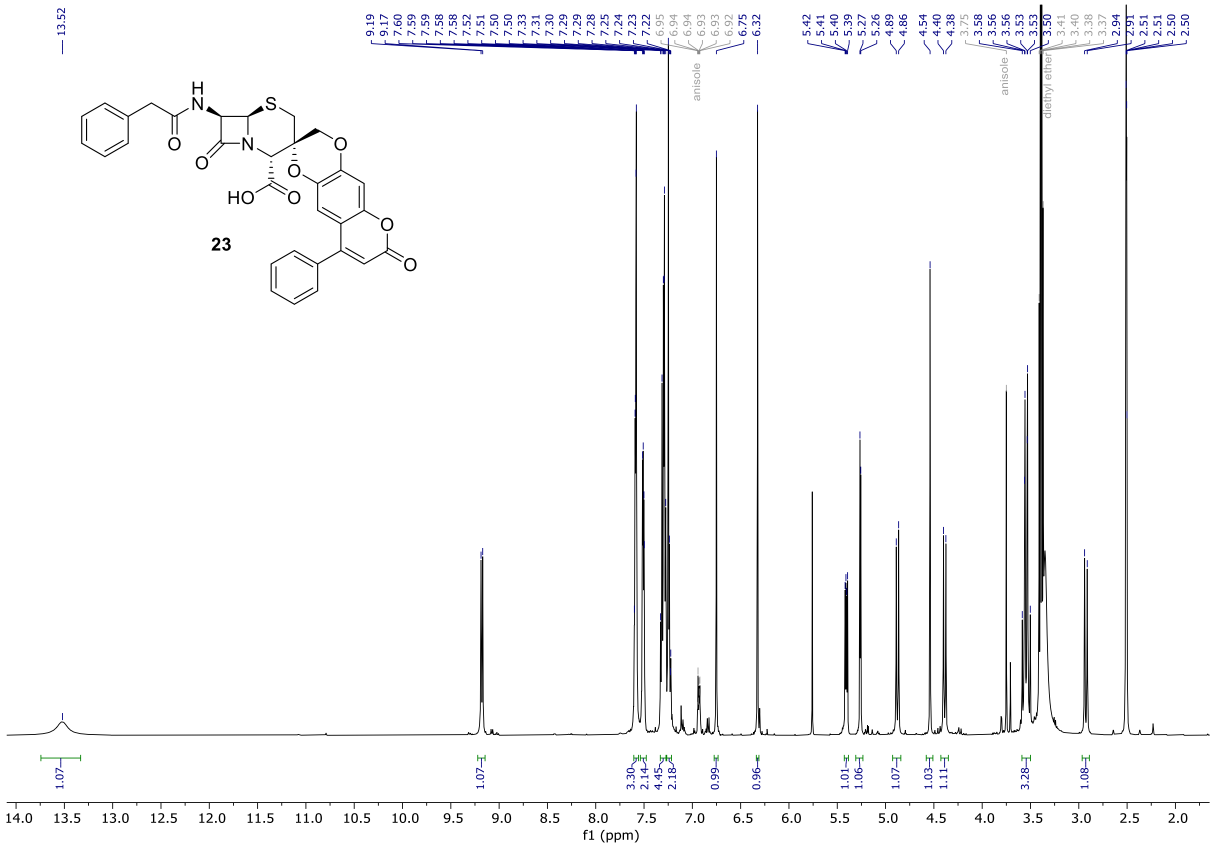

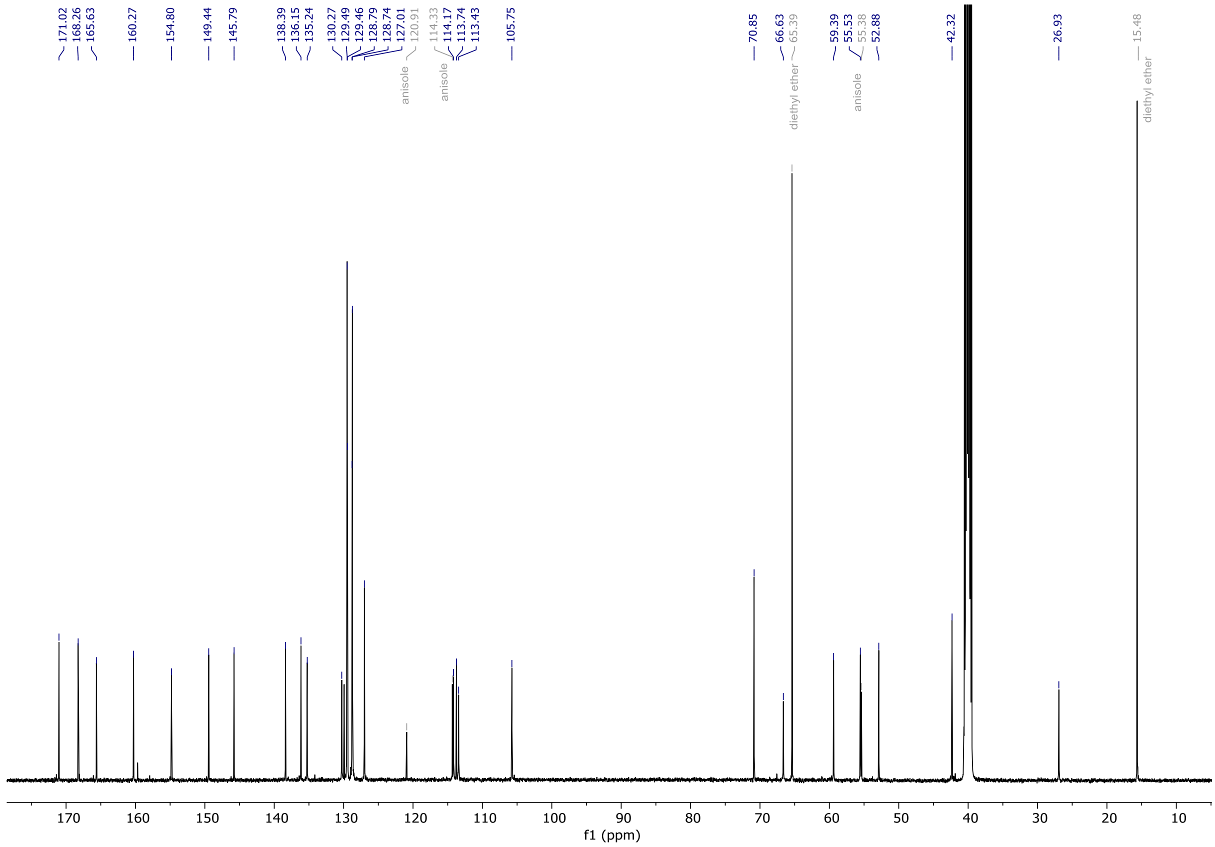

Figure S11. ^{1}H (top) and ^{13}C NMR (bottom) spectra of spiro-compound **23**.

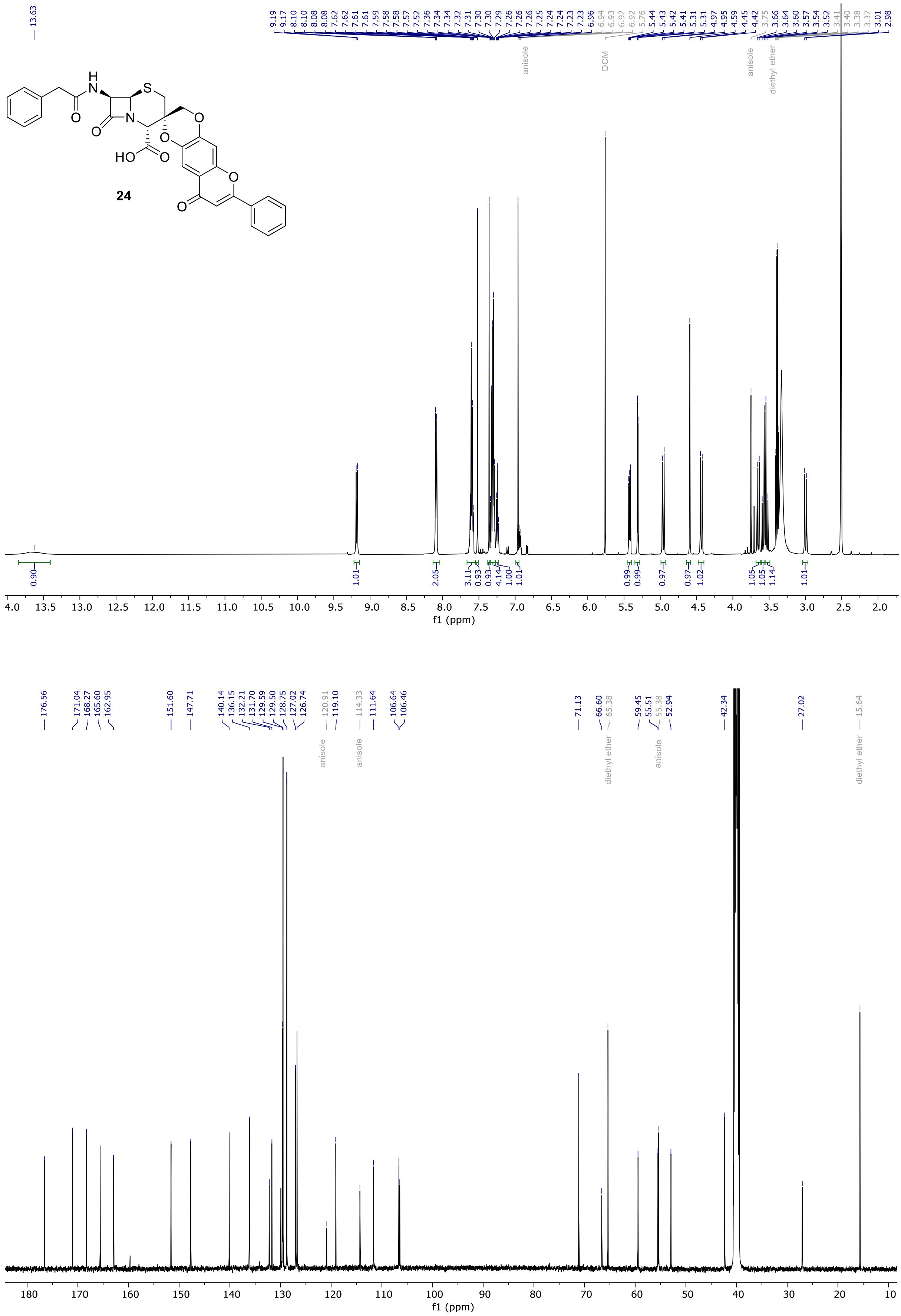

Figure S12. ^{1}H (top) and ^{13}C NMR (bottom) spectra of spiro-compound **24**.

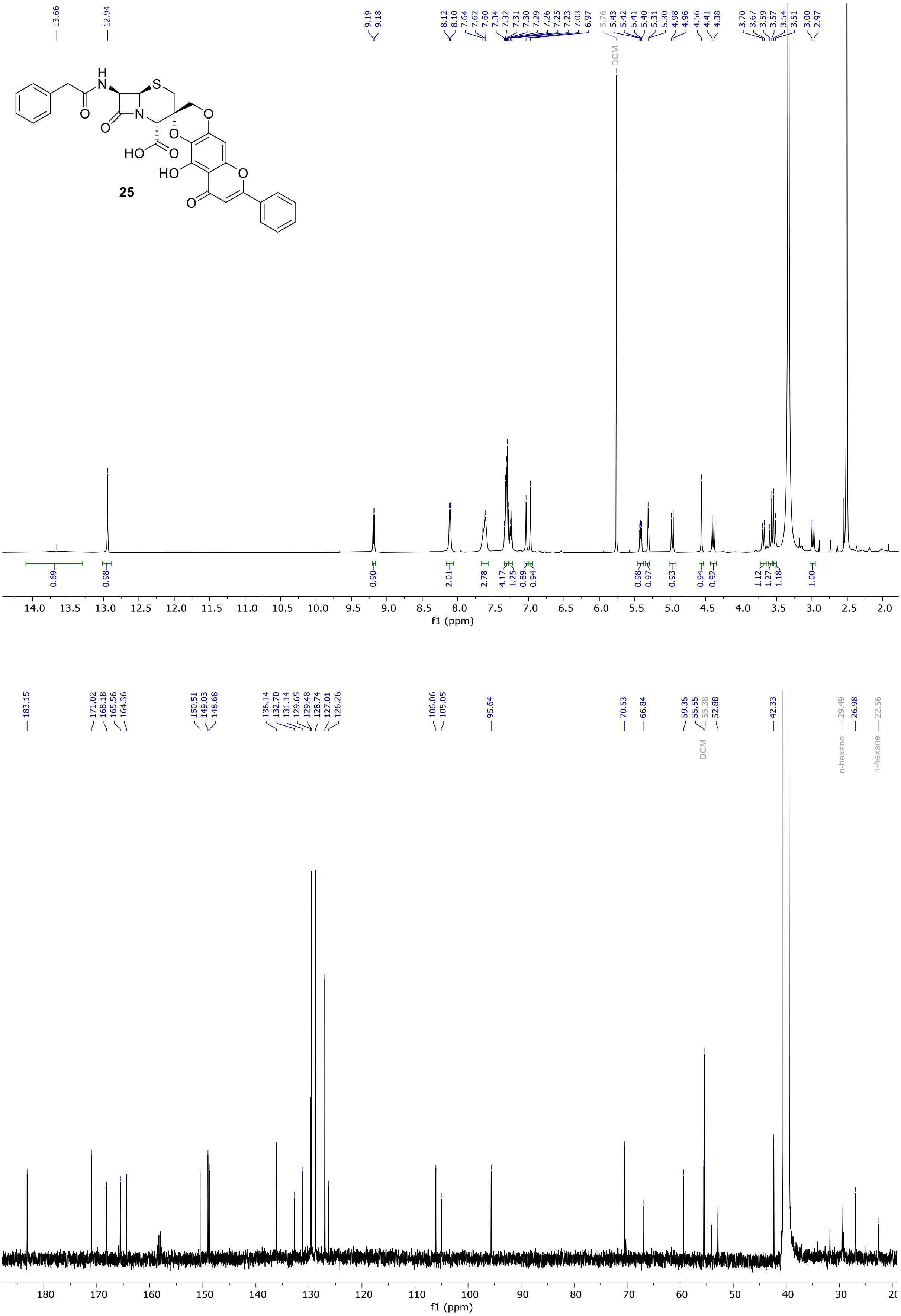

Figure S13. ^{1}H (top) and ^{13}C NMR (bottom) spectra of spiro-compound **25**.

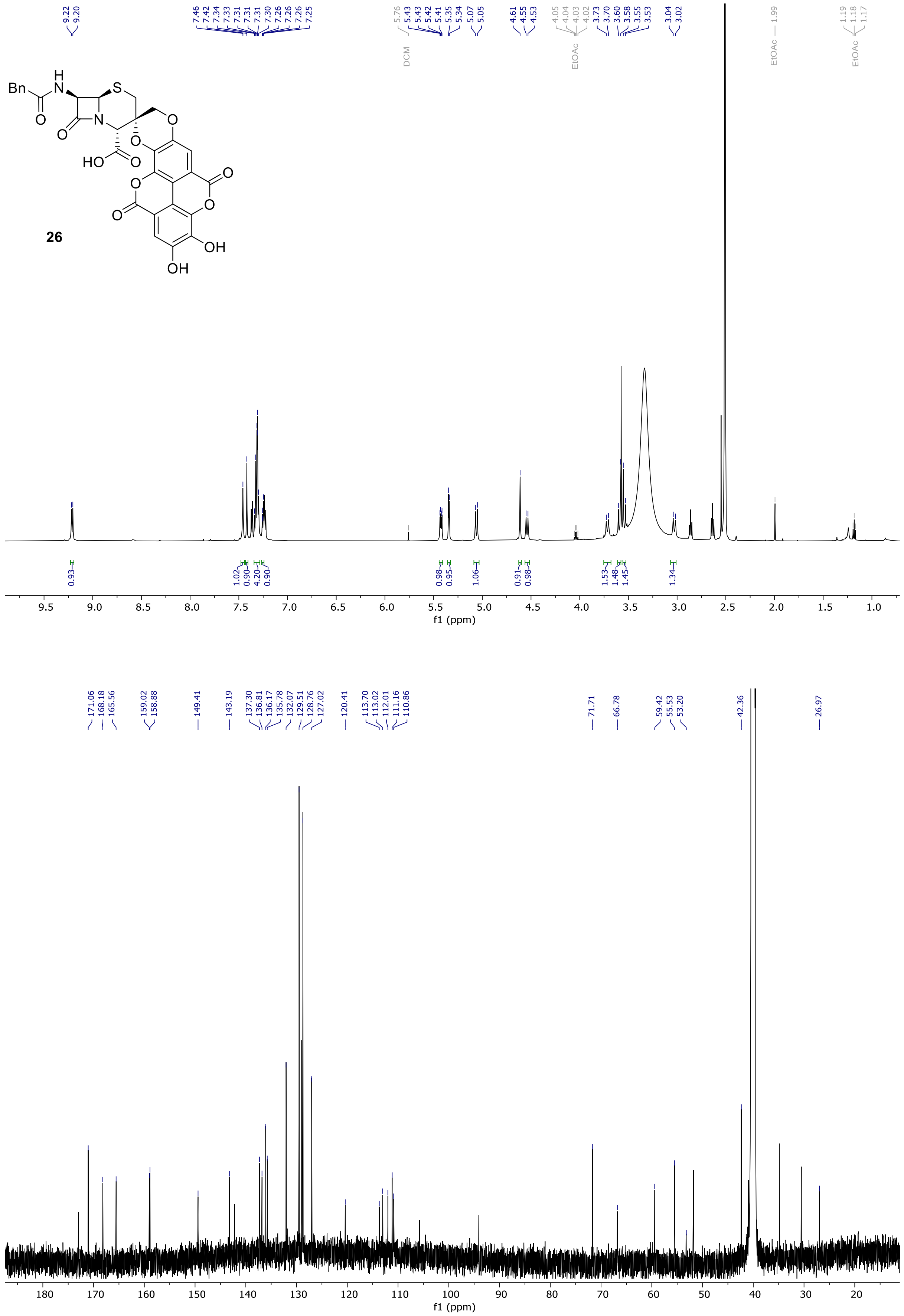

Figure S14. ^1H (top) and ^{13}C NMR (bottom) spectra of spiro-compound **26**.
